# Direct Observation of Transition Metal Ions Evolving into Single Atoms: Formation and Transformation of Nanoparticle Intermediates

**DOI:** 10.1002/advs.202206166

**Published:** 2023-03-02

**Authors:** Zheng Han, Yi Wang, Jiming Zheng, Ren Li, Boqian Jia, Dingding Li, Lei Bai, Xuting Guo, Lirong Zheng, Jinbo Bai, Kunyue Leng, Yunteng Qu

**Affiliations:** ^1^ State Key Laboratory of Photoelectric Technology and Functional Materials International Collaborative Center on Photoelectric Technology and Nano Functional Materials Institute of Photonics & Photon‐Technology Northwest University Xi'an 710069 P. R. China; ^2^ Institute of High Energy Physics Beijing 100049 P. R. China; ^3^ CentraleSupélec ENS Paris‐Saclay CNRS LMPS‐Laboratoire de Mécanique Paris‐Saclay Université Paris‐Saclay 8‐10 rue Joliot‐Curie Gif‐sur‐Yvette 91190 France

**Keywords:** nanoparticles, single atom catalysts, transition metals

## Abstract

Understanding the dynamical evolution from metal ions to single atoms is of great importance to the rational development of synthesis strategies for single atom catalysts (SACs) against metal sintering during pyrolysis. Herein, an in situ observation is disclosed that the formation of SACs is ascertained as a two‐step process. There is initially metal sintering into nanoparticles (NPs) (500–600 °C), followed by the conversion of NPs into metal single atoms (Fe, Co, Ni, Cu SAs) at higher temperature (700–800 °C). Theoretical calculations together with control experiments based on Cu unveil that the ion‐to‐NP conversion can arise from the carbon reduction, and NP‐to‐SA conversion being steered by generating more thermodynamically stable Cu‐N_4_ configuration instead of Cu NPs. Based on the evidenced mechanism, a two‐step pyrolysis strategy to access Cu SACs is developed, which exhibits excellent ORR performance.

## Graphical abstract

Given the beneficial properties of single atom catalysts (SACs), understanding their evolution from metal ions is critical. This present work demonstrates the direct observation of transition metal ions evolving into SACs with a detailed explanation. Specifically, metals are aggregated into nanoparticles at lower temperatures (500–600 °C), and subsequently transformed into metal single atoms at higher temperatures (700–800 °C).

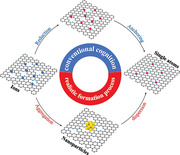

## 1 Introduction

**Scheme 1:**
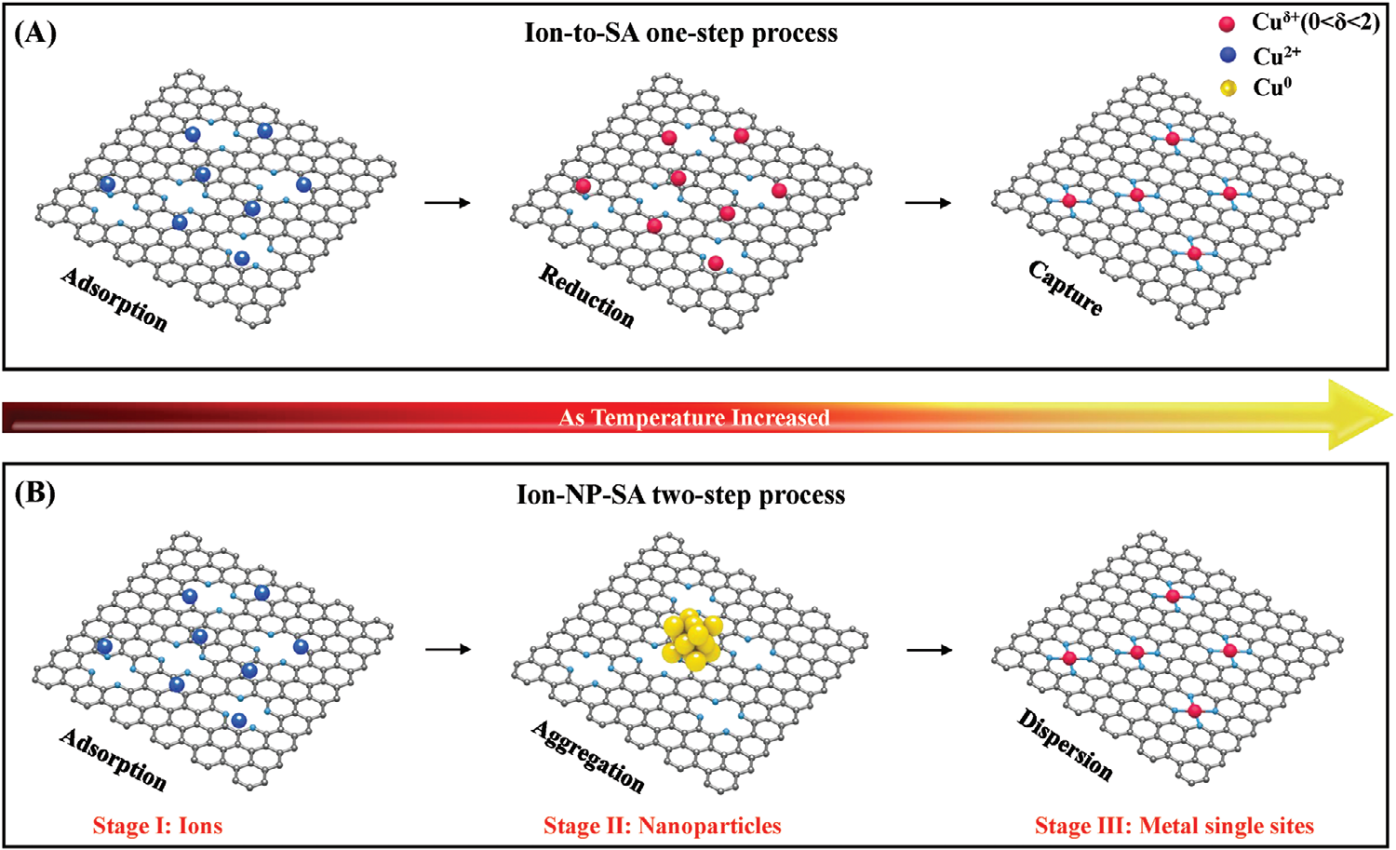
Schematic illustration of SACs formation process. A) traditional ion‐to‐SA one‐step process, B) practical ion‐NP‐SA two‐step process.

Nitrogen coordination transition metal single atom catalysts supported on carbon matrix (SACs‐NC) feature the maximum atomic utilization and unique electronic structure,^[^ ^1^, ^2^, ^3^ ^]^ and show great potential in fields of energy conversion and chemical synthesis.^[^ ^4^, ^5^, ^6^, ^7^, ^8^, ^9^, ^10^, ^11^, ^12^ ^]^ In order to achieve its promising application, splendid efforts had been made in developing synthetic strategy for well‐defined SACs‐NC, especially the strategy for large‐scale preparation, but still challenging.^[^ ^13^, ^14^, ^15^, ^16^, ^17^, ^18^, ^19^ ^]^ Of the various pioneering innovations, the strategy combining metal ion impregnation and annealing (MIA) is considered as the approach that most commonly used and most amenable to scale‐up due to its easy operation and low cost.^[^ ^20^, ^21^, ^22^, ^23^, ^24^, ^25^, ^26^, ^27^ ^]^ To further optimize the MIA strategy, the formation process of SACs‐NC had also been investigated. Traditionally, metal ions are thought to suffer from the adsorption, reduction, followed by anchoring on defects of supports, that is the metal ions directly transformed into SACs without additional steps (**Scheme** **1**A).^[^ ^21^, ^28^, ^29^, ^30^, ^31^ ^]^ However, it is still fuzzy whether a metal single atom directly forms via metal ions with one‐ or multiple‐step process.^[^ ^32^ ^]^ The knowledge gap generally generated undesired metal clusters or nanoparticles (NPs), particularly at elevated temperature or introducing high metal contents.^[^ ^1^, ^33^, ^34^, ^35^, ^36^ ^]^ This observation not only enables the loss of active sites, but also causes problems in accurately modulating coordination environment.^[^ ^20^, ^37^, ^38^, ^39^, ^40^, ^41^, ^42^ ^]^ A molecular level understanding that how metal ions evolved into SACs‐NC could provide important guidance to circumvent the metal sintering, in particular when scale‐up preparation is practiced. However, the dynamic evolution process of metal atomization from ions remains obscure and is rarely discussed.

In this work, we disclosed an unconventional two‐step process for the formation of SACs‐NC under the MIA approach based on the experimental investigation and DFT calculation. The metal ions initially underwent a sintering process into metal NPs, followed by the conversion of metal NPs into metal single atoms (Fe, Co, Ni, Cu) at higher temperature (Scheme 1B). We select nitrogen doped carbon (NC) derived by ZIF‐8 or guanine as the substrate materials to trap metal atoms. According to the operando TEM and XRD results, the obvious Cu NPs can be observed at the temperature range from 500 to 600 °C. Then, the Cu NPs gradually disappeared and transformed into metal single atoms at higher temperature (700–800 °C). Interestingly, when the pure carbon (XC‐72 and graphene) was adopted to replace NC as substrate materials, the Cu NPs would only grow steadily into larger Cu NPs as the temperature increased to 800 °C. This observation indicates the formation and enlargement of metal NPs can be attributed to the reducing capacity of carbon, while the nitrogen defect sites was closely related with transforming metal NPs into single atoms. Furthermore, based on the ascertained mechanism, we develop a two‐step pyrolysis strategy to prepare Cu SACs, which exhibited excellent ORR performance.

## 2 Results and Discussion

### 2.1 Cu Ion‐NP‐SA Evolution Process

**Figure 1:**
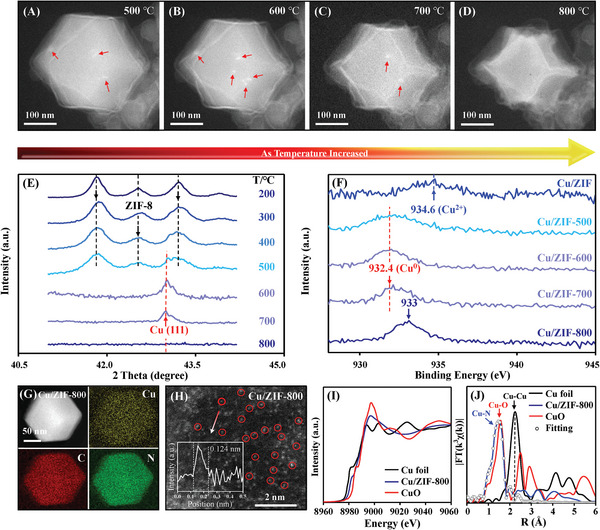
Cu ion‐NP‐SA two‐step evolution process during annealing. A–D) In situ ETEM images of Cu/ZIF from 500 to 800 °C, dark field. E) In situ XRD patterns of Cu/ZIF from 200 to 800 °C. F) Cu 2p XPS spectra of Cu/ZIFs pyrolyzed at different temperatures. G) HAADF‐TEM image of Cu/ZIF‐800, coupled with EDS mappings. H) AC HAADF‐STEM image of Cu/ZIF‐800. Inset, corresponding intensity profile. I) Cu K edge X‐ray absorption near‐edge structure (XANES) spectrum of Cu/ZIF‐800. J) k^3^‐weighted *χ*(k) function of EXAFS spectrum of Cu/ZIF‐800.

A classical dual solvent ions adsorption‐pyrolysis strategy was used to prepare transition metal single atom catalysts (Fe, Co, Ni, Cu SACs, Figure S1, Supporting Information), in which the ZIF‐8 nanocrystal with the size around 200–300 nm was selected as the precursors of NC supports (Figure S2, Supporting Information). Initially, the ion‐NP‐SA conversion process was discovered on Cu SACs. We adopted in situ ETEM techniques to identify the atomization process at the temperature range from room temperature to 800 °C. As shown in **Figure** **1**A and Figure S3 (Supporting Information), the tiny Cu clusters and NPs can be observed at 500 °C due to the reduction and aggregation of Cu ions. When temperature increased to 600 °C, the dispersed Cu clusters grew into larger NPs (Figure 1B). Subsequently, the number of Cu NPs gradually decreased at 700 °C (Figure 1C) and then completely disappeared at 800 °C (Figure 1D). For excluding the effects of consecutive electron beam at operando conditions, ex situ TEM tests were also conducted, as shown in Figure S4 (Supporting Information). The Fast Fourier Transform (FFT) images and 3D atom‐overlapping Gaussian‐function fitting mapping of the TEM images clearly demonstrated the formation of crystalline Cu at 500 and 600 °C (Figure S4A,B, Supporting Information). The crystalline structure evolution of Cu was further confirmed by the in situ XRD patterns of Cu/ZIF (Figure 1E). A diffraction peak at about 43° attributed to Cu (111) plane appeared at 500–700 °C and disappeared at 800 °C, in line with the in situ ETEM results and ex situ XRD patterns (Figure S5, Supporting Information). Note that the ZIF‐8 still maintained initial structure before 600 °C, as further verified by Raman spectra (Figure S6, Supporting Information). No significant difference was observed on Cu/ZIF between 600 and 800 °C in XPS spectra of C 1s and N 1s (Figure S7, Supporting Information) and EPR spectra (Figure S8, Supporting Information). Furthermore, the Cu element presented zero valent state at 500–700 °C and subsequently transformed into Cu^*δ*+^ (0 < *δ* < 2) at 800 °C (Figure 1F).^[^ ^27^ ^]^ These results significantly demonstrated the formation and transformation of Cu NPs under pyrolysis process.

The resulted Cu/ZIF‐800 exhibited uniform element distribution of Cu, N, and C, according to EDS mapping analysis (Figure 1G). Aberration‐corrected HAADF‐STEM images showed that atomically dispersed Cu single atoms with a size of 1.24 Å can be discerned on supports (Figure 1H and Figure S9, Supporting Information).^[^ ^21^ ^]^ For further confirming the formation of Cu SACs, we conducted X‐ray absorption fine structure (XAFS) testing (Figure 1I,J). The white‐line intensity of Cu/ZIF‐800 located between Cu foil and CuO, indicating the partial oxidation state between Cu (0) and Cu (II).^[^ ^2^ ^]^ This observation was greatly consistent with the XPS analysis above. The XAFS curves at R‐space and corresponding fitting analysis showed that only one dominant peak at about 1.48 Å was detected, which can be attributed to the Cu‐N_4_ coordination structure (Figure 1J and Table S1, Supporting Information).^[^ ^26^, ^43^ ^]^ These results provided solid evidences in verifying the formation of atomically dispersed Cu single atoms. Together, the formation of Cu SACs was confirmed to go through ion‐NP‐SA two‐step process rather than direct ion‐to‐SA one‐step process. At higher temperature such as 1200 °C, Cu NPs can be formed again (Figure S10, Supporting Information), which might be associated with the elimination of N species (Figure S11, Supporting Information).

### 2.2 Evolution Process of Fe, Co, and Ni

**Figure 2:**
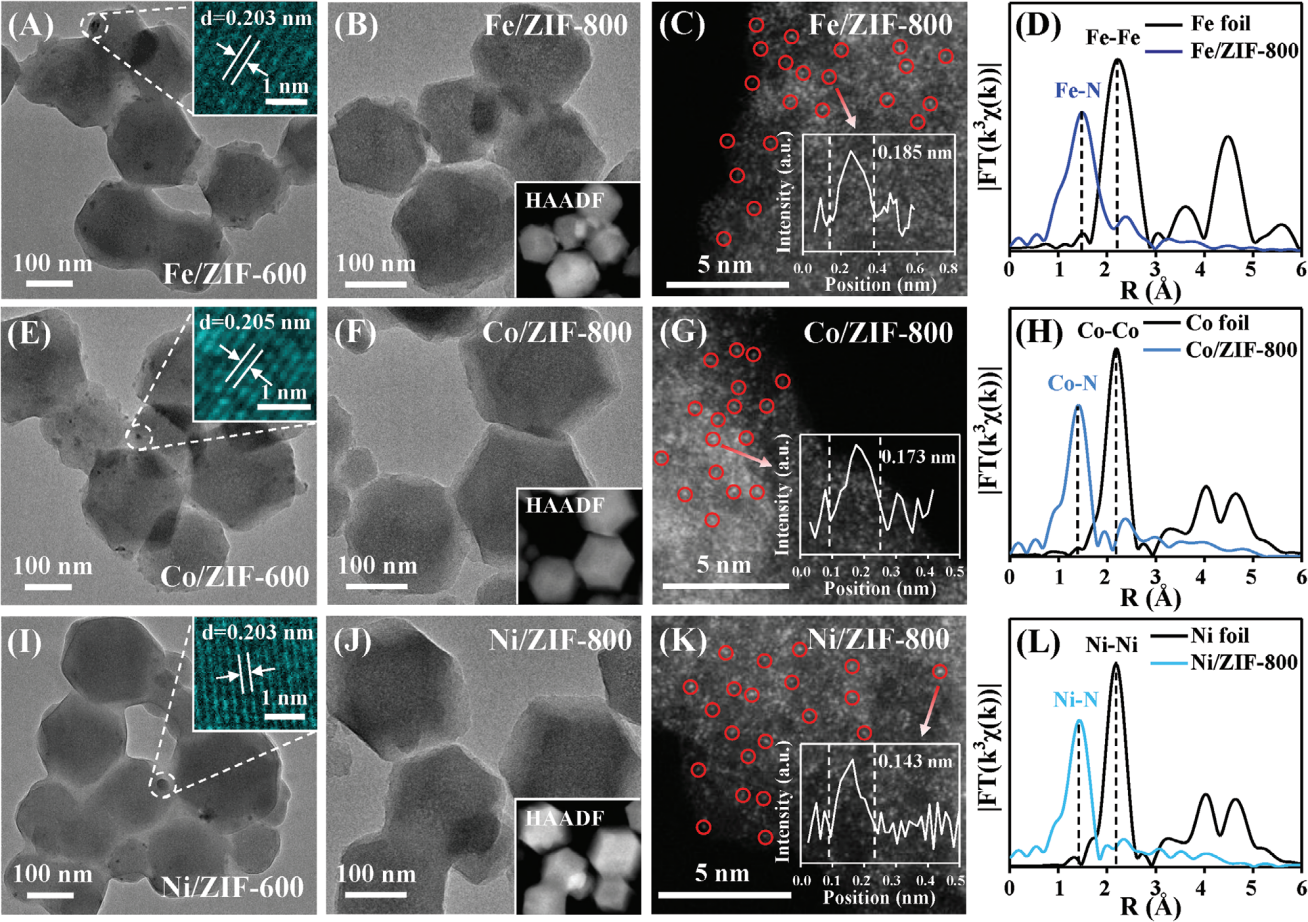
Evolution process of Fe, Co, and Ni during annealing. A,E,I) TEM images of Fe/ZIF‐600, Co/ZIF‐600, and Ni/ZIF‐600. Inset, HRTEM images of the black spots. B,F,J) TEM images of Fe/ZIF‐800, Co/ZIF‐800, and Ni/ZIF‐800. Inset, corresponding HAADF‐TEM images. C,G,K) AC HAADF‐STEM images of Fe/ZIF‐800, Co/ZIF‐800, and Ni/ZIF‐800. Inset, intensity profiles of the bright spots. k^3^‐weighted *χ*(k) function of EXAFS spectrum of D) Fe/ZIF‐800, H) Co/ZIF‐800, and L) Ni/ZIF‐800.

Inspired by the observation above, we also probed the dynamic evolution process of other transition metals, such as Fe, Co, and Ni, to testify the universality of ion‐NP‐SA two‐step conversion rules. As shown in **Figure** **2**A,E,I, the obvious Fe, Co, and Ni NPs was observed at 600 °C, as further attested by corresponding XRD patterns, SAED patterns and EDS mapping images (Figures S12–S14, Supporting Information). Then, the Fe, Co, and Ni NPs disappeared at 800 °C (Figure 2B,F,G). The as‐obtained Fe/ZIF‐800, Co/ZIF‐800, and Ni/ZIF‐800 showed uniformly dispersed Fe (Figure S15, Supporting Information), Co (Figure S16, Supporting Information), and Ni (Figure S17, Supporting Information) over whole architecture, confirming the existence of Fe, Co, and Ni in ultimate samples (pyrolysis at 800 °C). HAADF‐STEM images exhibited the Fe, Co, and Ni single atoms were homogeneously distributed on supports, indicating the transformation from NPs to SAs (Figure 2C,G,K and Figures S15–S17, Supporting Information). XAFS results demonstrated that only Fe—N, Co—N, and Ni—N bonds can be discerned at R‐space, further verifying the formation of Fe, Co, and Ni SACs (Figure 2D,H,L and Figures S18–S20, Supporting Information).^[^ ^29^, ^44^, ^45^ ^]^ These results indicated the two‐step ion‐NP‐SA evolution was not a unique observation but a universal phenomenon for MIA synthesis.

### 2.3 Mechanism Study Based on Carbon‐Based Supports

**Figure 3:**
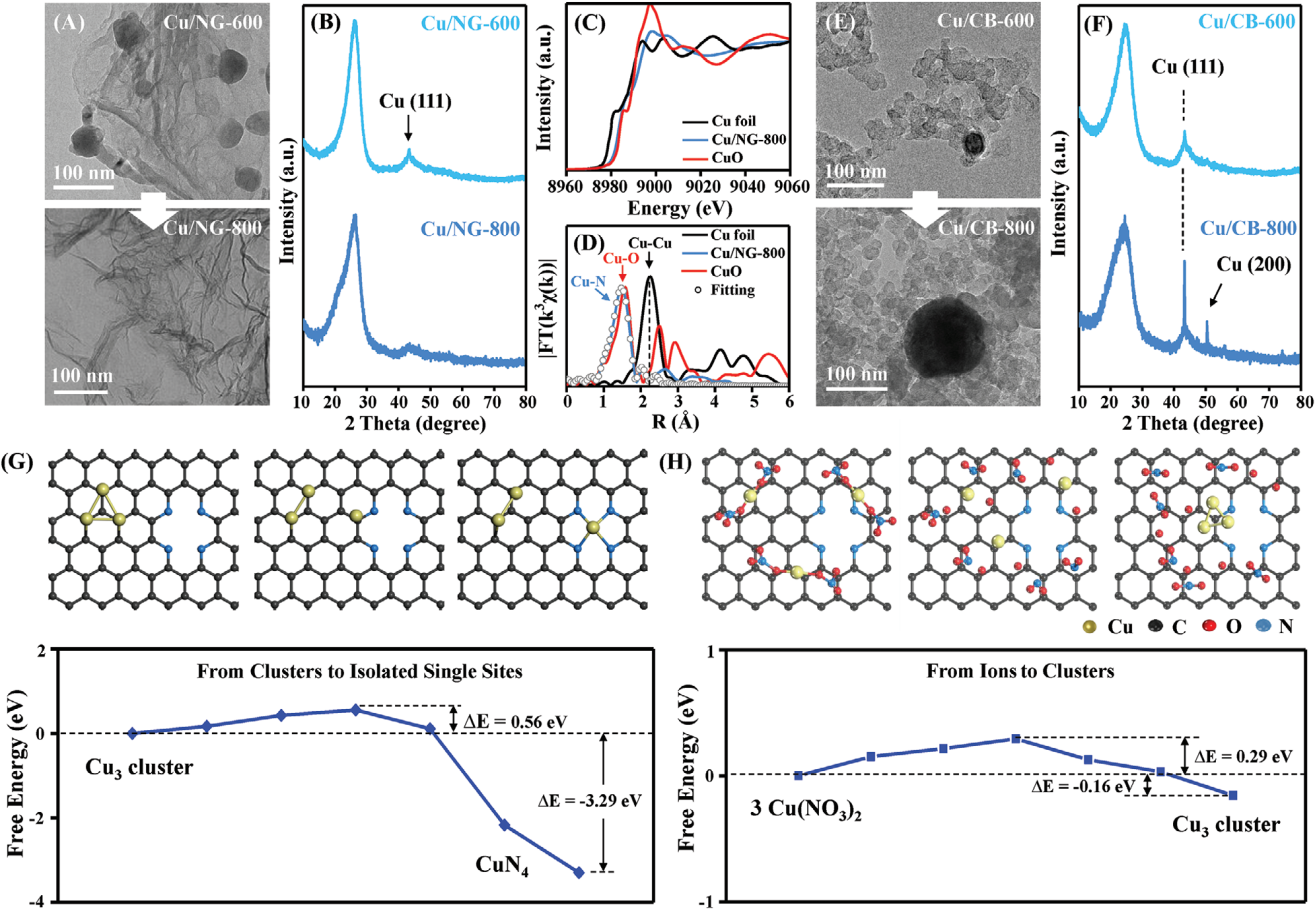
Theoretical studies based on graphene‐like N‐doped carbon (NG) and carbon black (CB). A) TEM images of Cu/NG‐600 and Gu/NG‐800. B) XRD patterns of Cu/NG‐600 and Cu/NG‐800. C) Cu K edge XANES spectrum of Cu/NG‐800. D) k^3^‐weighted *χ*(k) function of EXAFS spectrum of Cu/NG‐800. E) TEM images of Cu/CB‐600 and Gu/CB‐800. F) XRD patterns of Cu/CB‐600 and Cu/CB‐800. G) Evolution model from Cu_3_ cluster to CuN_4_ coordination structure (top), and the calculated energy diagram (bottom). H) evolution model from 3Cu(NO_3_)_2_ to Cu_3_ cluster (top), and the calculated energy diagram (bottom).

Considering the generation of plentiful volatilization species during ZIF‐8 pyrolysis (Figure S11, Supporting Information), prefabricated graphene‐like N‐doped carbon (NG) derived by guanine at 1000 °C was adopted as supports to revalidate the ion‐NP‐SA conversion. This prefabricated support exhibited high thermal stability at high temperature, as shown in the TG analysis (Figure S21A, Supporting Information). As shown in **Figure** **3**A–D and Figure S21 (Supporting Information), Cu ions still aggregated into Cu NPs at 600 °C (Cu/NG‐600), which was then transformed into Cu SAs at 800 °C (Cu/NG‐800), indicating the ion‐NP‐SA conversion was not determined by volatile species. Also, control experiments were carried out on N‐free carbon black XC‐72 (CB) and graphene (GP). Interestingly, the Cu ions were reduced to small Cu NPs at 600 °C and subsequently grew into larger Cu NPs at 800 °C (Figure 3E,F and Figure S22, Supporting Information), failing to transform Cu NPs to Cu SAs. On the basis of these results, one can reasonably speculate the formation and development of metal NPs can be attributed to the reducing capacity of carbon under thermal treatment, while the nitrogen defect sites play key effects on transforming metal NPs into SAs.

To further shed light on the mechanism, we constructed a typical Cu_3_ clusters to investigate the ion‐NP‐SA conversion process on NC supports (Figure 3G,H). The formation of Cu_3_ clusters from Cu ions and transformation of Cu_3_ clusters into CuN_4_ moieties showed obvious exothermicity of 0.16 and 3.29 eV, respectively. This indicated the whole ion‐NP‐SA conversion was a thermo‐spontaneous process. On the other hand, the NP‐to‐SA process exhibited a larger barrier of 0.56 eV to overcome than that of ion‐to‐NP process (0.29 eV). In this case, the ion‐to‐NP conversion became primary at relatively low‐temperature (≈500–600 °C) and NP‐to‐SA conversion dominated at high temperature (≈700–800 °C).

### 2.4 Application of Ion‐NP‐SA Process

**Figure 4:**
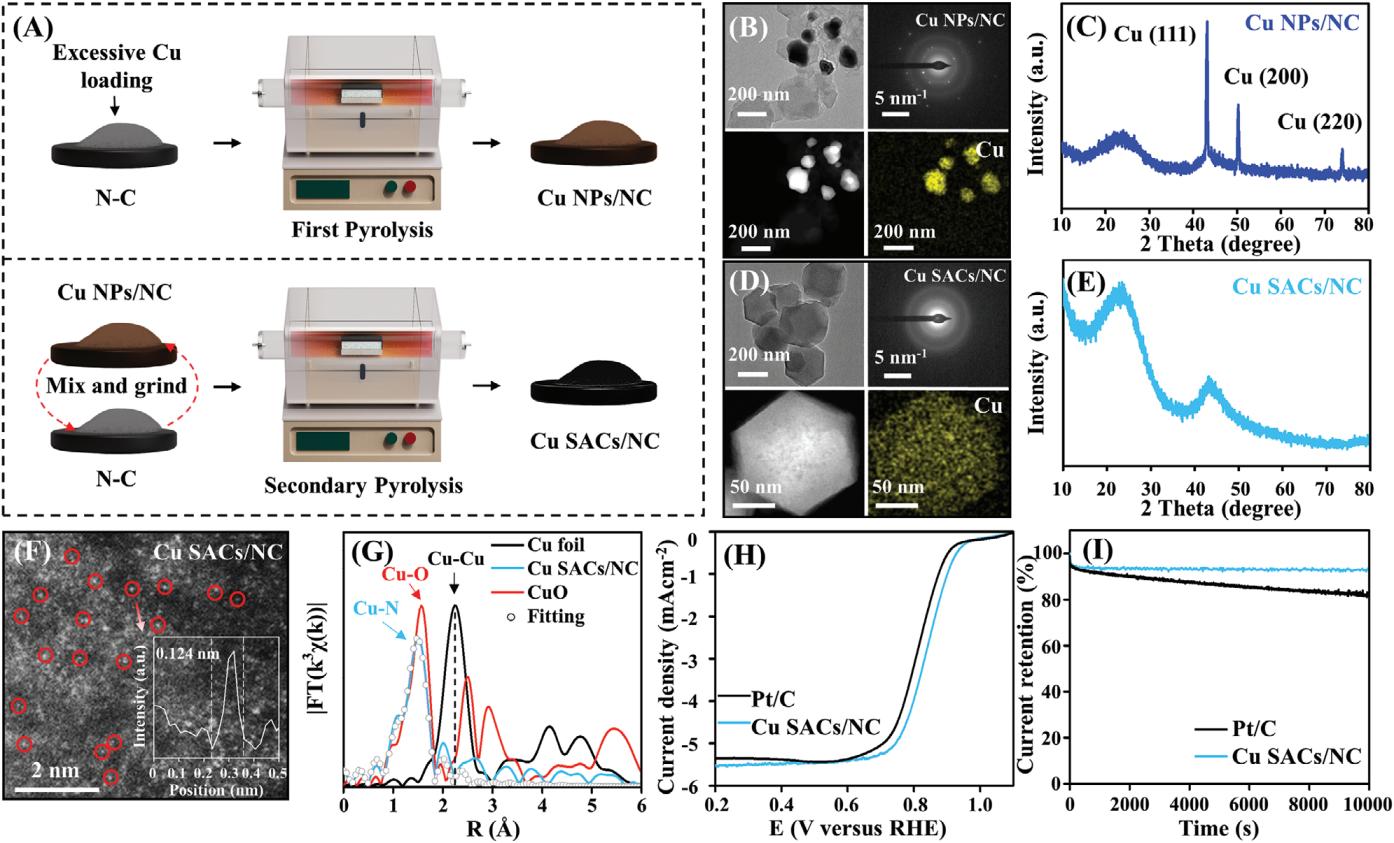
Application of the ion‐NP‐SA process. A) schematic illustration of two‐step pyrolysis strategy. B) TEM, SAED, and EDS mapping images of Cu NPs/NC. C) XRD pattern of Cu NPs/NC. D) TEM, SAED, and EDS mapping images of Cu SACs/NC. E) XRD pattern of Cu SACs/NC. F) AC HAADF‐STEM image of Cu SACs/NC. G) k^3^‐weighted *χ*(k) function of EXAFS spectrum of Cu SACs/NC. H) Polarization curves of Pt/C and Cu SACs/NC in ORR application. I) The *i*–*t* chronoamperometric response of Pt/C and Cu SACs/NC.

In view of the discovery of ion‐NP‐SA process, the formation of SACs under MIA was originated from the thermal migration of metal atoms. Therefore, we designed a two‐step pyrolysis strategy to access SACs, as shown in **Figure** **4**A. Firstly, excessive Cu precursors were impregnated on N‐doped carbon in order to generate plenty of Cu NPs at 900 °C (denoted as Cu NPs/NC), as characterized by TEM (Figure 4B) and XRD (Figure 4C). Subsequently, extra N‐doped carbon was mixed with Cu NPs/NC and then calcined at 900 °C. After 2 h under the thermal treatment, smaller Cu particles than that in Cu NPs/NC were observed (Cu NP/NC‐2 h, Figure S23, Supporting Information), indicating the dispersion of Cu particles during the two‐step pyrolysis. Figure 4D,E shows the TEM and XRD of the obtained sample after 4 h of calcination (Cu SACs/NC), in which Cu NPs were not observed. As shown in Figure 4F, heavier isolated Cu atoms can be observed in its aberration‐corrected HAADF‐STEM image. And, only the Cu—N bond was detected in its XAFS curve, which strongly proved the formation of Cu SACs (Figure 4G and Figure S24, Supporting Information). The unsaturated Cu–N coordination (≈CuN_3_) was demonstrated by the detailed fitting results of the Cu K edge EXAFS (Table S1, Supporting Information).

The catalytic performance of Cu SACs/NC was evaluated in the oxygen reduction reaction (ORR). As a result, Cu SACs/NC exhibited excellent ORR activity with a half‐wave (E_1/2_) value of 0.87 V, 50 and 70 mV higher than that of Pt/C and Cu NPs/NC (Figure 4H and Figure S25, Supporting Information). The rotating ring‐disk electrode (RRDE) test revealed the near four electron ORR pathway and low H_2_O_2_ yields for Cu SACs/NC (Figure S26, Supporting Information). The robust stability of Cu SACs/NC was confirmed by the long‐term test (Figure 4I). Furthermore, the XRD pattern, TEM image and EXAFS spectrum of the used catalyst indicated the maintaining of the structure of N coordinated Cu SACs (Figure S27, Supporting Information). Based on the structure identification, contrast ORR experiment of Cu NP and previously splendid works,^[^ ^46^, ^47^, ^48^ ^]^ the excellent ORR performance of Cu SACs/NC can be attributed to its unsaturated Cu‐N coordination single atom sites, which enables the modulation of ORR dynamics over Cu.

## 3 Conclusion

In summary, we discovered the ion‐NP‐SA evolution process of transition metals (Fe, Co, Ni, Cu) during ions impregnation‐pyrolysis synthesis. The formation of SACs supported on NC was ascertained as a two‐step process, that was initially metal sintering into NPs (500–600 °C), followed by the conversion of NPs into metal single atoms (Fe, Co, Ni, Cu SAs) at higher temperature (700–800 °C). This discovery reveals the formation and transformation of metal NPs during the formation of SACs, which will provide valuable guidance to rationally develop synthesis strategies of SACs.

## 4 Experimental Section

### Preparation of M/ZIFs (M = Fe, Co, Ni, Cu)

100 mg of ZIF‐8 powder was dispersed in 10 mL n‐hexane under ultrasound for 15 min at room temperature. Then, 50 µL of 0.4 m metal nitrate aqueous solution (Fe(NO_3_)_3_·9H_2_O, Co(NO_3_)_2_·6H_2_O, Ni(NO_3_)_2_·6H_2_O or Cu(NO_3_)_2_·3H_2_O) was added into the above solution with stirring for 6 h. After centrifugation and stoving, the obtained powders were labeled as M/ZIF. The pyrolyzed M/ZIFs were denoted as M/ZIF‐X, where X corresponded to the pyrolysis temperatures.

### Preparation of NG and Cu/NG

The powder of guanine was calcined at 1000 °C for 2 h under argon atmosphere (denoted as NG). 100 mg of NG powder was dispersed in 10 mL n‐hexane under ultrasound for 15 min at room temperature. Then, 25 µL of 0.4 m Cu(NO_3_)_2_·3H_2_O aqueous solution was added into the above solution under ultrasound for 6 h. The mixed suspension was centrifuged and dried in vacuum at 80 °C. The obtained Cu/NG was calcined at 600 and 800 °C for 1 h under argon atmosphere, respectively (labeled as Cu/NG‐600 and Cu/NG‐800).

### Preparation of Cu/CB

100 mg of XC‐72 (CB) powder was dispersed in 10 mL n‐hexane under ultrasound for 15 min at room temperature. Then, 25 µL of 0.4 m Cu(NO_3_)_2_·3H_2_O aqueous solution was added into the above solution under ultrasound for 6 h. The mixed suspension was centrifuged and dried in vacuum at 80 °C. Next, the Cu/CB was calcined at 600 and 800 °C for 1 h under argon atmosphere, respectively (labeled as Cu/CB‐600 and Cu/CB‐800).

### Preparation of Cu NPs/NC and Cu SACs/NC

The NC (N‐doped carbon) support was acquired by pyrolyzing ZIF‐8 at 900 °C for 1 h under argon atmosphere. Then, 0.4 m Cu(NO_3_)_2_·3H_2_O aqueous solution was added in NC support, to prepare the Cu/NC mixture with 15 wt% Cu loading. After impregnation and maturation, the prepared sample was dried at 60 °C under vacuum overnight, which was then calcined at 900 °C for 1 h under argon atmosphere and denoted as Cu NPs/NC. Next, 3 g NC powder and 1 g Cu NPs/NC were mixed and ground. This mixture was calcined at 900 °C for 4 h under argon atmosphere (labeled as Cu SACs/NC).

## Conflict of Interest

The authors declare no conflict of interest.

## Supporting information

Supporting Information

## Data Availability

The data that support the findings of this study are available in the supplementary material of this article.

## References

[1] S. Ji , Y. Chen , X. Wang , Z. Zhang , D. Wang , Y. Li , Chem. Rev. 2020, 120, 11900. 32242408 10.1021/acs.chemrev.9b00818

[2] Y. Qu , Z. Li , W. Chen , Y. Lin , T. Yuan , Z. Yang , C. Zhao , J. Wang , C. Zhao , X. Wang , F. Zhou , Z. Zhuang , Y. Wu , Y. Li , Nat. Catal. 2018, 1, 781.

[3] J. Gu , M. Jian , L. Huang , Z. Sun , A. Li , Y. Pan , J. Yang , W. Wen , W. Zhou , Y. Lin , H.‐J. Wang , X. Liu , L. Wang , X. Shi , X. Huang , L. Cao , S. Chen , X. Zheng , H. Pan , J. Zhu , S. Wei , W.‐X. Li , J. Lu , Nat. Nanotechnol. 2021, 16, 1141. 34312515 10.1038/s41565-021-00951-y

[4] X. Wu , Q. Wang , S. Yang , J. Zhang , Y. Cheng , H. Tang , L. Ma , X. Min , C. Tang , S. P. Jiang , F. Wu , Y. Lei , S. Ciampic , S. Wang , L. Dai , Energy Environ. Sci. 2022, 15, 1183.

[5] W. Liu , L. Zhang , X. Liu , X. Yang , S. Miao , W. Wang , A. Wang , T. Zhang , J. Am. Chem. Soc. 2017, 139, 10790. 28745500 10.1021/jacs.7b05130

[6] H. B. Yang , S.‐F. Hung , S. Liu , K. Yuan , S. Miao , L. Zhang , X. Huang , H.‐Y. Huang , W. Cai , R. Chen , J. Gao , X. Yang , W. Chen , Y. Huang , H. M. Chen , C. M. Li , T. Zhang , B. Liu , Nat. Energy 2018, 3, 140.

[7] Z.‐Y. Wu , M. Karamad , X. Yong , Q. Huang , D. A. Cullen , P. Zhu , C. Xia , Q. Xiao , M. Shakouri , F.‐Y. Chen , J. Y. Kim , Y. Xia , K. Heck , Y. Hu , M. S. Wong , Q. Li , I. Gates , S. Siahrostami , H. Wang , Nat. Commun. 2021, 12, 2870. 34001869 10.1038/s41467-021-23115-x PMC8128876

[8] Y. Cai , J. Fu , Y. Zhou , Y.‐C. Chang , Q. Min , J.‐J. Zhu , Y. Lin , W. Zhu , Nat. Commun. 2021, 12, 586. 33500393 10.1038/s41467-020-20769-x PMC7838205

[9] J. Li , M. Chen , D. A. Cullen , S. Hwang , M. Wang , B. Li , K. Liu , S. Karakalos , M. Lucero , H. Zhang , C. Lei , H. Xu , G. E. Sterbinsky , Z. Feng , D. Su , K. L. More , G. Wang , Z. Wang , G. Wu , Nat. Catal. 2018, 1, 935.

[10] F. Luo , A. Roy , L. Silvioli , D. A. Cullen , A. Zitolo , M. T. Sougrati , I. C. Oguz , T. Mineva , D. Teschner , S. Wagner , J. Wen , F. Dionigi , U. I. Kramm , J. Rossmeisl , F. Jaouen , P. Strasser , Nat. Mater. 2020, 19, 1215. 32661387 10.1038/s41563-020-0717-5

[11] L. Cao , Q. Luo , J. Chen , L. Wang , Y. Lin , H. Wang , X. Liu , X. Shen , W. Zhang , W. Liu , Z. Qi , Z. Jiang , J. Yang , T. Yao , Nat. Commun. 2019, 10, 4849. 31649237 10.1038/s41467-019-12886-z PMC6813412

[12] S. Fang , X. Zhu , X. Liu , J. Gu , W. Liu , D. Wang , W. Zhang , Y. Lin , J. Lu , S. Wei , Y. Li , T. Yao , Nat. Commun. 2020, 11, 1029. 32098951 10.1038/s41467-020-14848-2 PMC7042219

[13] X. Wang , S. Ding , T. Yue , Y. Zhu , M. Fang , X. Li , G. Xiao , Y. Zhu , L. Dai , Nano Energy 2021, 82, 105689.

[14] L. Zhao , Y. Zhang , L.‐B. Huang , X.‐Z. Liu , Q.‐H. Zhang , C. He , Z.‐Y. Wu , L.‐J. Zhang , J. Wu , W. Yang , L. Gu , J.‐S. Hu , L.‐J. Wan , Nat. Commun. 2019, 10, 1278. 30894539 10.1038/s41467-019-09290-y PMC6426845

[15] C. Xia , Y. Qiu , Y. Xia , P. Zhu , G. King , X. Zhang , Z. Wu , J. Y. Kim , D. A. Cullen , D. Zheng , P. Li , M. Shakouri , E. Heredia , P. Cui , H. N. Alshareef , Y. Hu , H. Wang , Nat. Chem. 2021, 13, 887. 34168326 10.1038/s41557-021-00734-x

[16] X. Hai , S. Xi , S. Mitchell , K. Harrath , H. Xu , D. F. Akl , D. Kong , J. Li , Z. Li , T. Sun , H. Yang , Y. Cui , C. Su , X. Zhao , J. Li , J. Pérez‐Ramírez , J. Lu , Nat. Nanotechnol. 2022, 17, 174. 34824400 10.1038/s41565-021-01022-y

[17] H. Yang , L. Shang , Q. Zhang , R. Shi , G. I. N. Waterhouse , L. Gu , T. Zhang , Nat. Commun. 2019, 10, 4585. 31594928 10.1038/s41467-019-12510-0 PMC6783464

[18] Y. Zhao , Z. Pei , X. Lu , D. Luan , X. Wang , X. W. Lou , Chem. Catal. 2022, 2, 1480.

[19] X. Liu , C. Ao , X. Shen , L. Wang , S. Wang , L. Cao , W. Zhang , J. Dong , J. Bao , T. Ding , L. Zhang , T. Yao , Nano Lett. 2020, 11, 8319. 10.1021/acs.nanolett.0c03475 33090809

[20] Y. Zhang , L. Jiao , W. Yang , C. Xie , H.‐L. Jiang , Angew. Chem., Int. Ed. 2021, 60, 7607. 10.1002/anie.202016219 33432715

[21] S. Ma , Z. Han , K. Leng , X. Liu , Y. Wang , Y. Qu , J. Bai , Small 2020, 16, 2001384. 10.1002/smll.202001384 32363699

[22] B. Jia , L. Bai , Z. Han , R. Li , J. Huangfu , C. Li , J. Zheng , Y. Qu , K. Leng , Y. Wang , J. Bai , ACS Appl. Mater. Interfaces 2022, 14, 10337. 35179878 10.1021/acsami.1c23079

[23] W. Ye , S. Chen , Y. Lin , L. Yang , S. Chen , X. Zheng , Z. Qi , C. Wang , R. Long , M. Chen , J. Zhu , P. Gao , L. Song , J. Jiang , Y. Xiong , Chem 2019, 5, 2865.

[24] Z. Li , Y. Chen , S. Ji , Y. Tang , W. Chen , A. Li , J. Zhao , Y. Xiong , Y. Wu , Y. Gong , T. Yao , W. Liu , L. Zheng , J. Dong , Y. Wang , Z. Zhuang , W. Xing , C.‐T. He , C. Peng , W.‐C. Cheong , Q. Li , M. Zhang , Z. Chen , N. Fu , X. Gao , W. Zhu , J. Wan , J. Zhang , L. Gu , S. Wei , et al., Nat. Chem. 2020, 12, 764. 32541950 10.1038/s41557-020-0473-9

[25] Y.‐N. Gong , L. Jiao , Y. Qian , C.‐Y. Pan , L. Zheng , X. Cai , B. Liu , S.‐H. Yu , H.‐L. Jiang , Angew. Chem., Int. Ed. 2020, 59, 2705. 10.1002/anie.201914977 31821685

[26] J. Yang , W. Liu , M. Xu , X. Liu , H. Qi , L. Zhang , X. Yang , S. Niu , D. Zhou , Y. Liu , Y. Su , J.‐F. Li , Z.‐Q. Tian , W. Zhou , A. Wang , T. Zhang , J. Am. Chem. Soc. 2021, 143, 14530. 34464109 10.1021/jacs.1c03788

[27] R. Jiang , L. Li , T. Sheng , G. Hu , Y. Chen , L. Wang , J. Am. Chem. Soc. 2018, 140, 11594. 30168714 10.1021/jacs.8b07294

[28] C. Zhao , X. Dai , T. Yao , W. Chen , X. Wang , J. Wang , J. Yang , S. Wei , Y. Wu , Y. Li , J. Am. Chem. Soc. 2017, 139, 8078. 28595012 10.1021/jacs.7b02736

[29] J. Wang , Z. Huang , W. Liu , C. Chang , H. Tang , Z. Li , W. Chen , C. Jia , T. Yao , S. Wei , Y. Wu , Y. Li , J. Am. Chem. Soc. 2017, 139, 17281. 29135246 10.1021/jacs.7b10385

[30] P. Yin , T. Yao , Y. Wu , L. Zheng , Y. Lin , W. Liu , H. Ju , J. Zhu , X. Hong , Z. Deng , G. Zhou , S. Wei , Y. Li , Angew. Chem., Int. Ed. 2016, 55, 10800. 10.1002/anie.201604802 27491018

[31] X. Guo , G. Fang , G. Li , H. Ma , H. Fan , L. Yu , C. Ma , X. Wu , D. Deng , M. Wei , D. Tan , R. Si , S. Zhang , J. Li , L. Sun , Z. Tang , X. Pan , X. Bao , Science 2014, 344, 616. 24812398 10.1126/science.1253150

[32] L. Zhang , Y. Li , L. Zhang , K. Wang , Y. Li , L. Wang , X. Zhang , F. Yang , Z. Zheng , Adv. Sci. 2022, 9, 2200592. 10.1002/advs.202200592 PMC9284138 35508897

[33] S. Wei , A. Li , J.‐C. Liu , Z. Li , W. Chen , Y. Gong , Q. Zhang , W.‐C. Cheong , Y. Wang , L. Zheng , H. Xiao , C. Chen , D. Wang , Q. Peng , L. Gu , X. Han , J. Li , Y. Li , Nat. Nanotechnol. 2018, 13, 856. 30013217 10.1038/s41565-018-0197-9

[34] Z. Li , S. Ji , Y. Liu , X. Cao , S. Tian , Y. Chen , Z. Niu , Y. Li , Chem. Rev. 2020, 120, 626. 10.1021/acs.chemrev.9b00311 31868347

[35] H. Huang , D. Yu , F. Hu , S.‐C. Huang , J. Song , H.‐Y. Chen , L. Li , S. Peng , Angew. Chem., Int. Ed. 2022, 134, e202116068. 10.1002/anie.202116068 34957659

[36] Z. Ma , S. Liu , N. Tang , T. Song , L. Motokura , Z. Shen , Y. Yang , ACS Catal. 2022, 12, 5595.

[37] A. K. Datye , Q. Xu , K. C. Kharas , J. M. McCarty , Catal. Today 2006, 111, 59.

[38] H. Zhou , Y. Zhao , J. Xu , H. Sun , Z. Li , W. Liu , T. Yuan , W. Liu , X. Wang , W.‐C. Cheong , Z. Wang , X. Wang , C. Zhao , Y. Yao , W. Wang , F. Zhou , M. Chen , B. Jin , R. Sun , J. Liu , X. Hong , T. Yao , S. Wei , J. Luo , Y. Wu , Nat. Commun. 2020, 11, 335. 31953446 10.1038/s41467-019-14223-w PMC6969067

[39] J. Zhang , Y. Zhao , C. Chen , Y.‐C. Huang , C.‐L. Dong , C.‐J. Chen , R.‐S. Liu , C. Wang , K. Yan , Y. Li , G. Wang , J. Am. Chem. Soc. 2019, 141, 20118. 31804069 10.1021/jacs.9b09352

[40] J. He , N. Li , Z.‐G. Li , M. Zhong , Z.‐X. Fu , M. Liu , J.‐C. Yin , Z. Shen , W. Li , J. Zhang , Z. Chang , X.‐H. Bu , Adv. Funct. Mater. 2021, 31, 2103597.

[41] C. Tang , Y. Jiao , B. Shi , J.‐N. Liu , Z. Xie , X. Chen , Q. Zhang , S.‐Z. Qiao , Angew. Chem., Int. Ed. 2020, 132, 9256. 10.1002/anie.202003842 32196867

[42] A. M. Abdel‐Mageed , B. Rungtaweevoranit , M. Parlinska‐Wojtan , X. Pei , O. M. Yaghi , R. J. Behm , J. Am. Chem. Soc. 2019, 141, 5201. 30852893 10.1021/jacs.8b11386

[43] Z. Yang , B. Chen , W. Chen , Y. Qu , F. Zhou , C. Zhao , Q. Xu , Q. Zhang , X. Duan , Y. Wu , Nat. Commun. 2019, 10, 3734. 31427572 10.1038/s41467-019-11796-4 PMC6700197

[44] W. Wan , Y. Zhao , S. Wei , C. A. Triana , J. Li , A. Arcifa , C. S. Allen , R. Cao , G. R. Patzke , Nat. Commun. 2021, 12, 5589. 34552084 10.1038/s41467-021-25811-0 PMC8458471

[45] Y. Chen , S. Ji , Y. Wang , J. Dong , W. Chen , Z. Li , R. Shen , L. Zheng , Z. Zhuang , D. Wang , Y. Li , Angew. Chem., Int. Ed. 2017, 56, 6937. 10.1002/anie.201702473 28402604

[46] L. Peng , J. Yang , Y. Yang , F. Qian , Q. Wang , D. Sun‐Waterhouse , L. Shang , T. Zhang , G. I. N. Waterhouse , Adv. Mater. 2022, 34, 2202544. 10.1002/adma.202202544 35584394

[47] X. Wang , Z. Chen , Z. Han , H. Gai , J. Zhou , Y. Wang , P. Cui , J. Ge , W. Xing , X. Zheng , M. Huang , H. Jiang , Adv. Funct. Mater. 2022, 32, 2111835.

[48] C.‐X. Zhao , B.‐Q. Li , J.‐N. Liu , Q. Zhang , Angew. Chem., Int. Ed. 2021, 60, 4448. 10.1002/anie.202003917 32315106

