# Supplementary material for: Direct Observation of Transition Metal Ions Evolving into Single Atoms: Formation and Transformation of Nanoparticle Intermediates
Source: Adv Sci (Weinh). 2023 Mar 2;10(12):2206166. doi: 10.1002/advs.202206166 (PMC10131801; doi:10.1002/advs.202206166)
Supplement: Supplementary file 1 — Supporting Information [file ADVS-10-2206166-s001.pdf]

ADVANCED SCIENCE
Open Access

Supporting Information

for *Adv. Sci.*, DOI 10.1002/advs.202206166

Direct Observation of Transition Metal Ions Evolving into Single Atoms: Formation and Transformation of Nanoparticle Intermediates

Zheng Han, Yi Wang, Jiming Zheng, Ren Li, Boqian Jia, Dingding Li, Lei Bai, Xuting Guo, Lirong Zheng, Jinbo Bai, Kunyue Leng* and Yunteng Qu**

Supporting Information

Direct Observation of Transition Metal Ions Evolving into Single Atoms: Formation and Transformation of Nanoparticle Intermediates

Zheng Han[a], Yi Wang*[a], Jiming Zheng[a], Ren Li[a], Boqian Jia[a], Dingding Li[a], Lei Bai[a], Xuting Guo[a], Lirong Zheng[b], Jinbo Bai[c], Kunyue Leng*[a] and Yunteng Qu*[a]

1. Experimental Procedures

Materials:

Iron nitrate nonahydrate ($Fe(NO_3)_3{\cdot}9H_2O$), Cobalt nitrate hexahydrate ($Co(NO_3)_2{\cdot}6H_2O$), Nickel nitrate hexahydrate ($Ni(NO_3)_2{\cdot}6H_2O$), Copper nitrate trihydrate ($Cu(NO_3)_2{\cdot}3H_2O$) and Guanine were purchased from Alfa Aesar Chemical Co. Carbon black (Vulcan XC-72) was purchased from Carbot Corporation. Methanol and Hexane were purchased from Shanghai Chemical Reagents. Graphene was purchased from Xiamen Knano company. The commercial Pt/C catalysts contains ~3 nm platinum nanoparticles with 20 wt%. All aqueous solutions were prepared with deionized (DI) water from ion-exchange and filtration system. All chemicals were used without further purification in this experiment.

Characterizations:

XRD patterns. In-situ XRD patterns were collected on Rigaku SmartLab, with a scan rate of 0.4 °/min between the 2θ range of 41 and 44.2 °. The test was carried out from 200 to 800 °C with a heating rate of 5 °C /min, under argon atmosphere. The ex-situ XRD patterns were performed on Bruker D8 advance diffractometer using Cu Kα radiation (λ = 0.15418 nm) operating at 40 kV.

XPS spectra. X-ray photoelectron spectra were collected on a PHI-5400 photoelectron spectrometer with Al Kα X-rays operating at 12.5 W. The spectra were collected with a pass energy of 280 eV for survey and 112 eV for scan. The spectra were analyzed using CasaXPS software and calibrated by the C 1s spectrum (284.5 eV).

Raman spectra. Raman spectra were detected on a Nicolet Almega dispersive Raman spectrometer with laser excitation at 532 nm.

TG analysis. Thermogravimetric analysis was carried out using a Pyris 1 TGA coupled with a PerkinElmer Clarus SQ 8 mass spectrometer at a heating rate of 10 °C/min in an inert atmosphere.

EPR spectra. Electron paramagnetic resonance spectra were recorded using the Bruker A200 spectrometer (Karlsruhe, Germany).

Electron microscopy characterization. Scanning electron microscopy (SEM) was carried out using a JEOL JSM-6390A instrument. Transmission electron microscopy (TEM), high-resolution TEM (HRTEM) images, the corresponding energy-dispersive X-ray spectroscopy (EDS) and selected area electron diffraction (SAED) were taken on a FEI Talos F200X. Aberration-corrected HAADF-STEM images were performed on a JEOL JEMARM200F TEM/STEM system. In-situ ETEM experiments were carried out on Thermo Scientific Themis G^3 ETEM equipped with DENS solutions Climate holder and Gas Supply System, using the primary electron energy of 300 keV. The sample were dispersed in alcohol and loaded in the MEMS-based nanoreactor, which was then mounted in a Climate holder. The environment inside was 1 bar He, heating by the MEMS heater.

XAFS measurements. The X-ray absorption fine structure spectra were collected at the Beijing Synchrotron Radiation Facility (BSRF) in China. The storage rings of BSRF were operated at 2.5 GeV with an average current of 250 mA. Using Si (111) double crystal monochromator, the data collection was carried out in transmission/fluorescence mode using ionization chamber. All spectra were collected in ambient conditions.

The acquired EXAFS data were processed according to the standard procedures using the ATHENA module implemented in the IFEFFIT software packages. The k^3-weighted EXAFS spectra were obtained by subtracting the post-edge background from the overall absorption and the normalizing with respect to the edge-jump step. Subsequently, k^3-weight $\chi(k)$ data of K-edge were Fourier transformed to real (R) space using a hanning windows (dk=1.0 $Å^{-1}$) to separate the EXAFS contributions from different coordination shells. To obtain the quantitative structural parameter around central atoms, least-squares curve parameter fitting was performed using the ARTEMIS module of IFEFFIT software packages.

Electrochemical measurements:

All electrochemical measurements for ORR were conducted in a conventional standard three-electrode system at room temperature on an electrochemical workstation (CHI 760E, CH Instrument, Shanghai, China). A rotating-disk electrode (RDE) equipment was connected to the three-electrode system to test the ORR activity. A glassy carbon (GC) disk with diameter of 5 mm was used as working electrode. Graphite rod and Ag/AgCl (3M KCl) were applied as the counter and reference electrodes, respectively. In this work, catalyst inks were prepared as follows: Cu SAs/NC and Pt/C were prepared by ultrasonically dispersing 5 mg of catalyst in 1 mL solution (containing 490 μL of ethanol, 490 μL of water, and 20 μL of 5 wt% Nafion). And,

the working electrode was prepared by coating 5 μL catalyst ink on glassy carbon RDE for 4 times. Before the ORR tests, the electrolyte (0.1 M KOH solution) was filled with O_2 flow for 30 min to achieve the O_2 saturated solution. All potential values were normalized to the reversible hydrogen electrode (RHE) according to the Nernst equation:

$$E_{[RHE]} = E_{[Ag/AgCl]} + 0.197 + 0.059\ pH$$

The RDE tests were carried out at a sweep rate of 10 mV/s with different rotating speeds ranging from 900 to 2500 rpm. The electron transfer number was determined by Koutecky-Levich equation:

$$\frac{1}{J} = \frac{1}{J_L} + \frac{1}{J_K} = \frac{1}{B\omega^{1/2}} + \frac{1}{J_k}$$

$$B = 0.62nFC_0D_0^{\frac{2}{3}}V^{-\frac{1}{6}}$$

Where J is the measured current density, J_L and J_K are diffusion-limiting and kinetic densities, respectively, ω indicates the angular velocity of the disk, n denotes the transferred electron number, F is the Faraday constant (85484 C mol^{-1}), C_0 is the bulk concentration of O_2 (1.2×10^{-6} mol cm^{-3}), D_0 is the diffusion coefficient of O_2 (1.9×10^{-5} cm^2 s^{-1}) and V is the kinematic viscosity of the electrolyte (0.01 cm^2 s^{-1}). The stability tests were performed by current vs. time (i-t) chronoamperometric response.

The rotating ring-disk electrode (RRDE) test was conducted by LSV in the same potential range at a scan rate of 10 mV/s at 1600 rpm, while the ring disk was set to 1.2 V vs RHE. The hydrogen peroxide yield (H_2O_2 %) and the electron transfer number (n) were calculated using the following equations:

$$H_2O_2(\%) = 100\frac{2I_R/N}{I_D + (I_R/N)}$$

$$n = \frac{4I_D}{I_D + (I_R/N)}$$

Where I_D is the disk current, I_R is the ring current, N is the H_2O_2 collection coefficient at the ring and N = 0.4.

Computational Method：

We have employed the first-principles[1,2] to perform density functional theory (DFT) calculations within the generalized gradient approximation (GGA) using the Perdew-Burke-Ernzerhof (PBE)[3] formulation. The Brillouin zone integration was performed using 3×3×1 Monkhorst-Pack k point sampling for structures. The models were set in a 8×7 supercell and the thickness of vacuum layer was set as 15 Å to get rid of the influence from the virtual interlayer interaction. We have chosen the projected augmented wave (PAW) potentials[4,5] to

describe the ionic cores and take valence electrons into account using a plane wave basis set with a kinetic energy cutoff of 450 eV. Partial occupancies of the Kohn−Sham orbitals were allowed using the Gaussian smearing method and a width of 0.03 eV. The electronic energy was considered self-consistent when the energy change was smaller than 10^{-6} eV. A geometry optimization was considered convergent when the energy change was smaller than 0.05 eV $Å^{-1}$. Grimme's DFT-D3 methodology[6] was used to describe the dispersion interactions among all the atoms in adsorption models. The adsorption energies (Eads) were calculated as Eads= Ead/sub -Ead -Esub, where Ead/sub, Ead, and Esub are the total energies of the optimized adsorbate/substrate system, the adsorbate in the structure, and the clean substrate, respectively. What's more, M ions energies had been evaluated using the climbing nudged elastic band (CI-NEB) methods.

Statistical Analysis：

The data with a symbol of ± presented as mean ± standard deviation calculated on a minimum of three independent samples.

2. Figures and Table

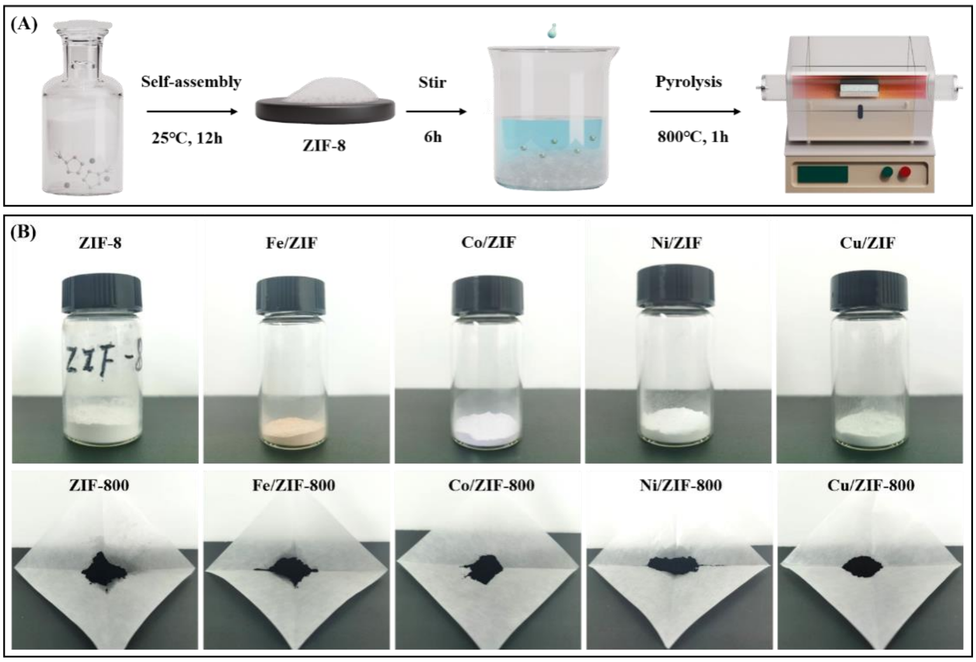

Figure S1. (A) Schematic illustration of the synthesis procedure. (B) Photographs of the prepared samples.

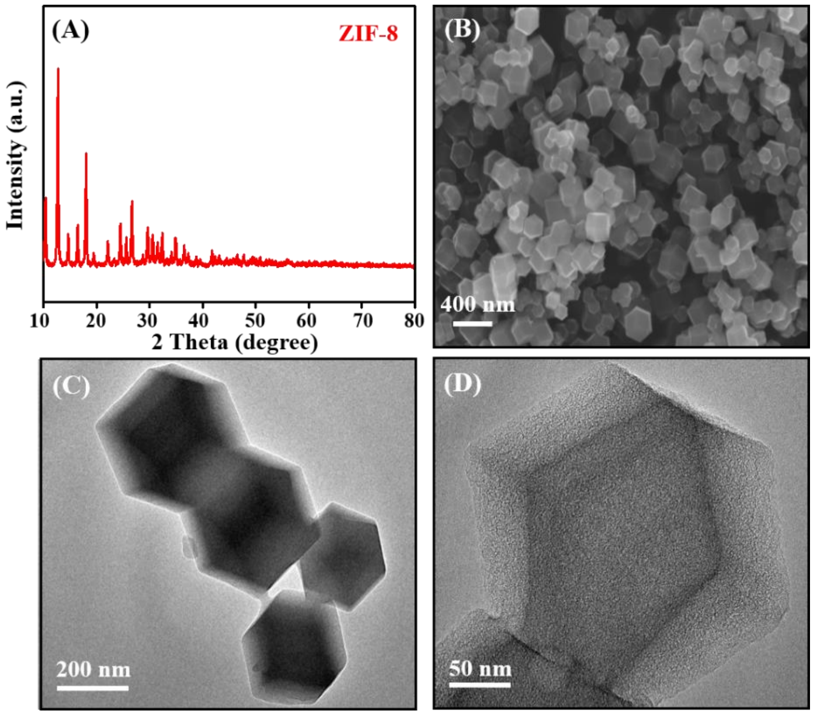

Figure S2. Characterizations of ZIF-8. (A) XRD. (B) SEM. (C, D) TEM.

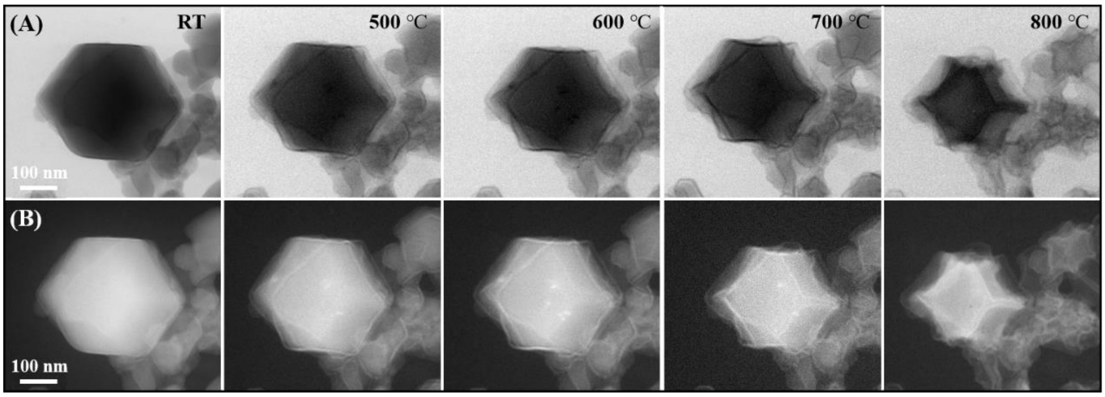

Figure S3. In-situ ETEM images of Cu/ZIF from RT to 800 °C. (A) Bright field images. (B) Dark field images.

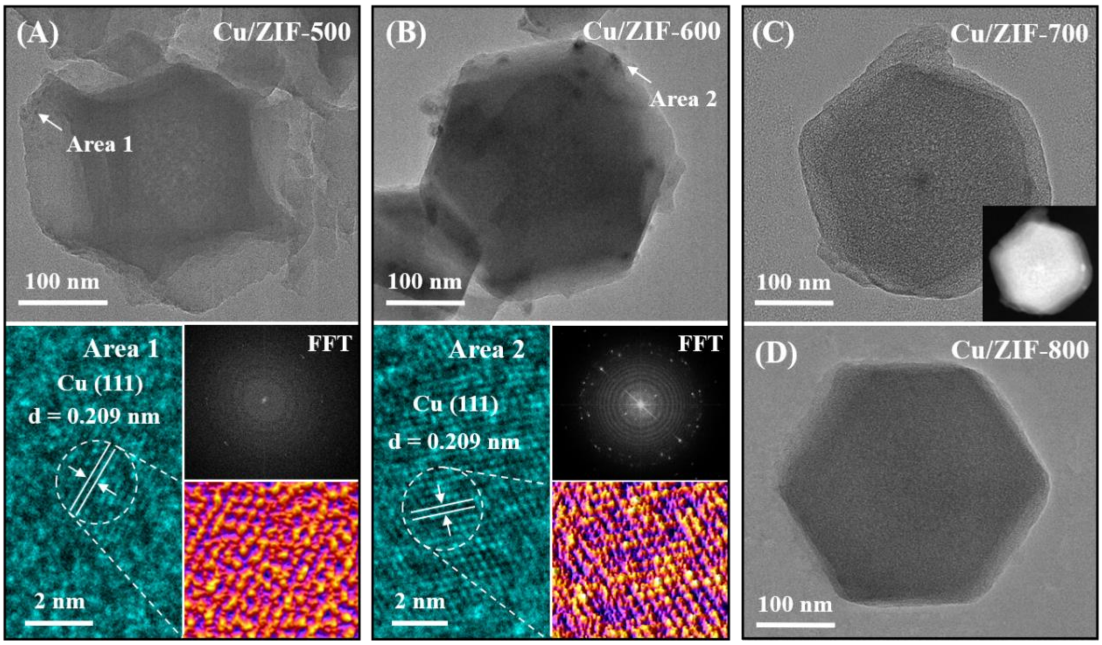

Figure S4. TEM images of pyrolyzed Cu/ZIF. (A, B) Cu/ZIF-500 and Cu/ZIF-600 (Top). Bottom, the corresponding HRTEM images, FFT images and 3D atom-overlapping Gaussian-function fitting mappings of the selected area. (C) Cu/ZIF-700. Inset, the corresponding HAADF-TEM. (D) Cu/ZIF-800.

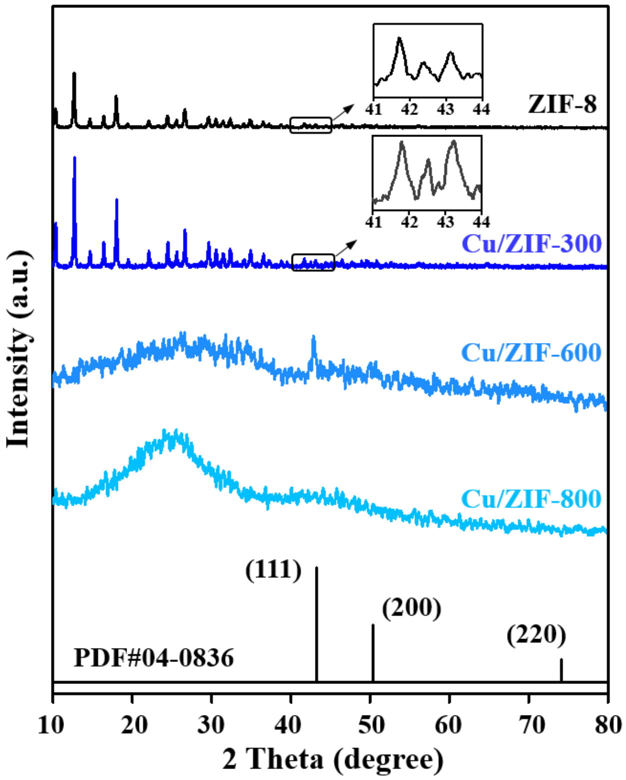

Figure S5. XRD patterns of pyrolyzed Cu/ZIFs. Inset shows the enlarged diffraction pattern between 41-44°.

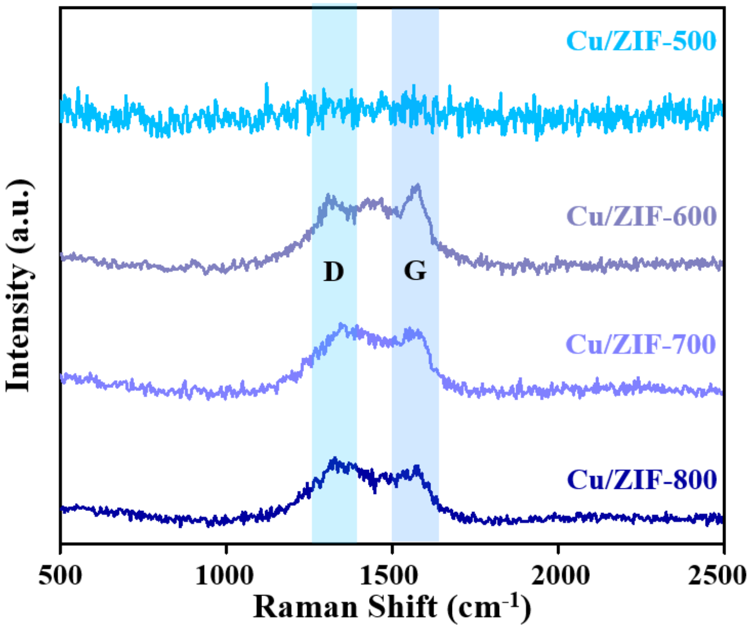

Figure S6. Raman spectra of pyrolyzed Cu/ZIFs.

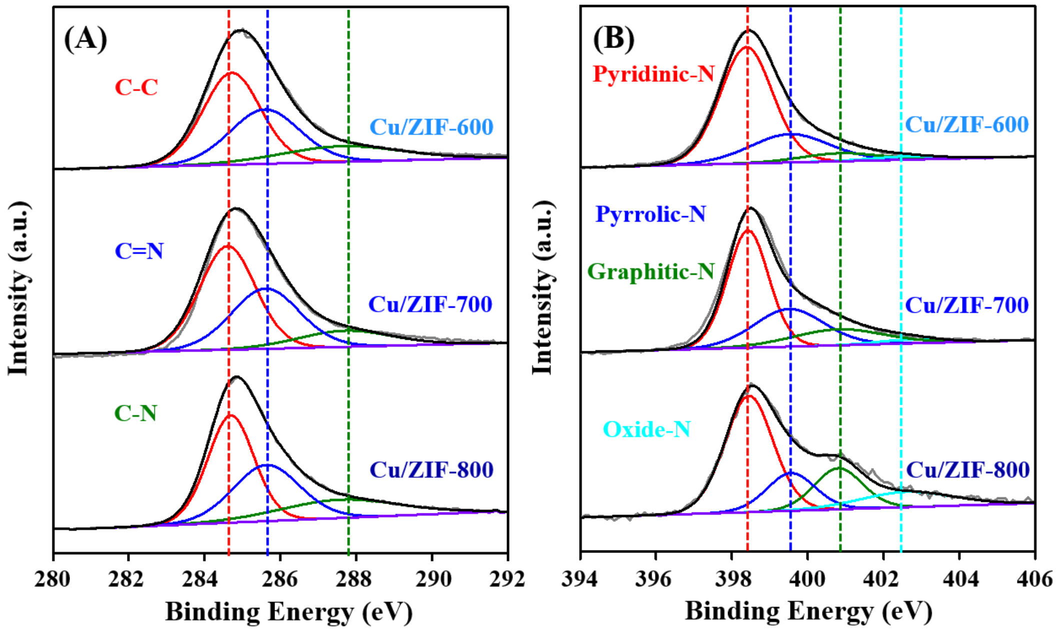

Figure S7. XPS spectra of Cu/ZIF-600, Cu/ZIF-700 and Cu/ZIF-800. (A) C 1s XPS spectra. (B) N 1s XPS spectra.

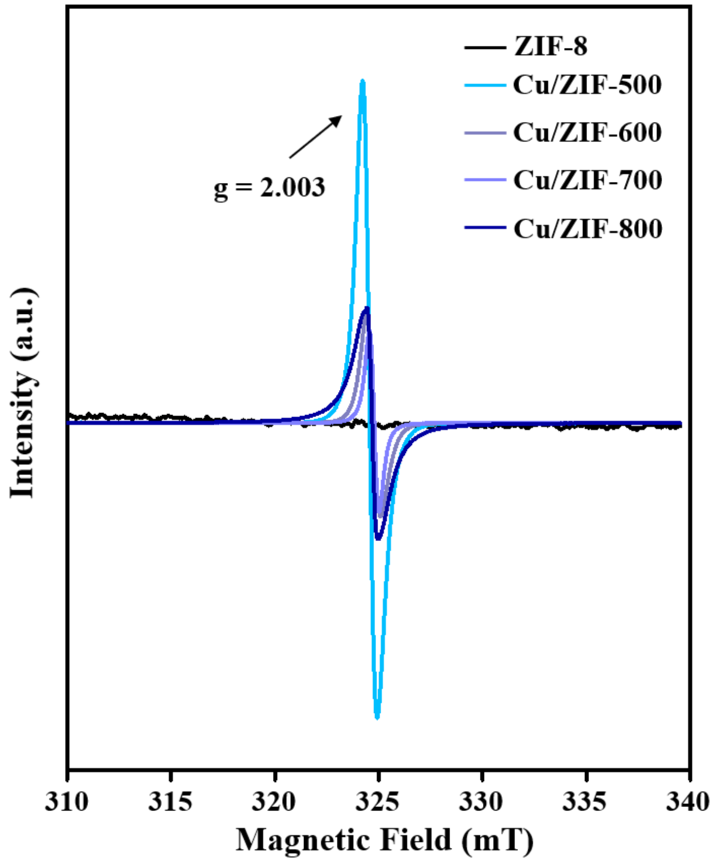

Figure S8. EPR spectra of Cu/ZIF-600, Cu/ZIF-700 and Cu/ZIF-800.

The vibration at g value of 2.003 is corresponding to the carbon radical signal. This signal is not observed in ZIF-8. For Cu/ZIF-500, this vibration become intense, reflecting the serious damage of ZIF-8 framework. For Cu/ZIF-600, Cu/ZIF-700 and Cu/ZIF-800, the EPR intensities are significantly decreased due to the formation of N-doped carbon.

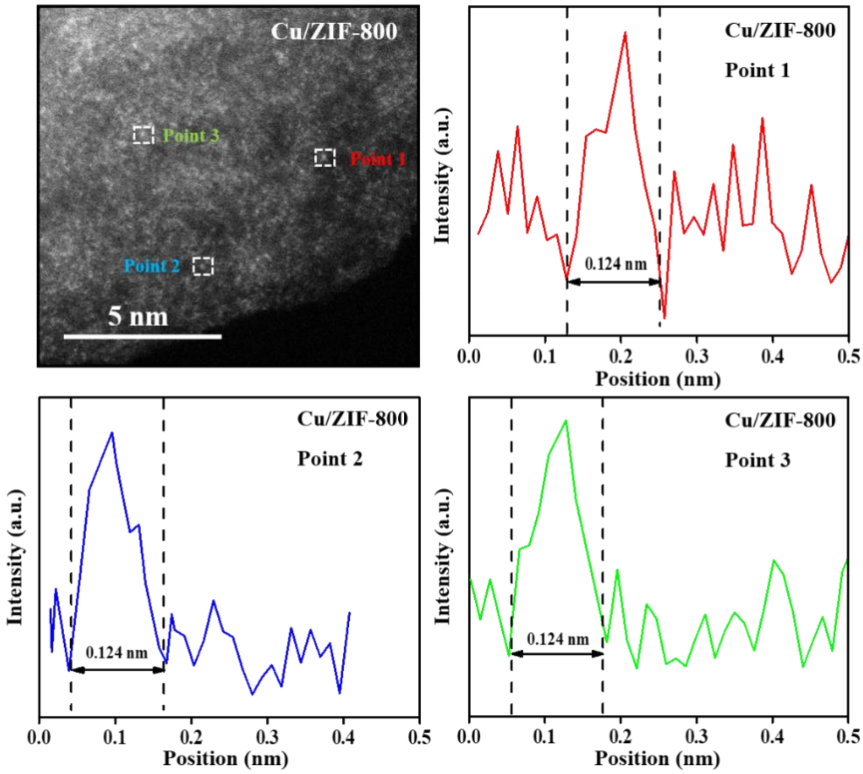

Figure S9. HAADF-STEM image of Cu/ZIF-800 and the corresponding intensity profiles of the selected bright spots.

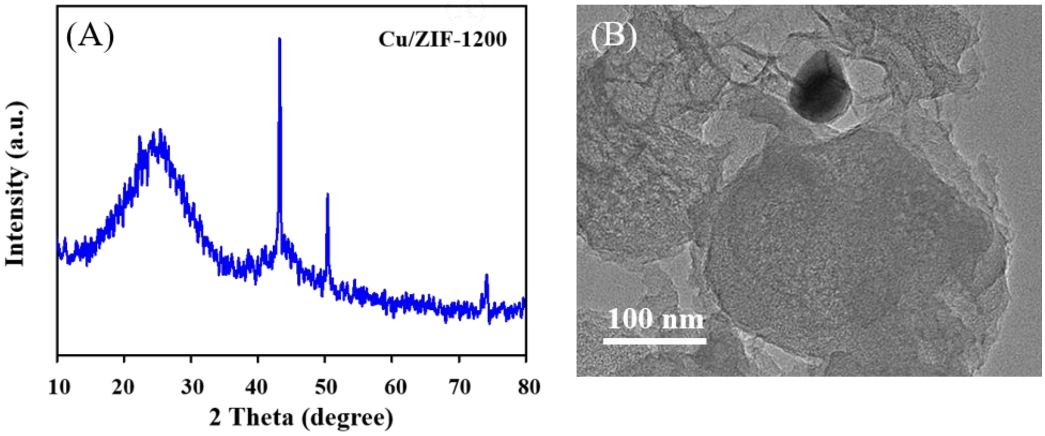

Figure S10. (A) XRD pattern of Cu/ZIF-1200. (B) TEM image of Cu/ZIF-1200.

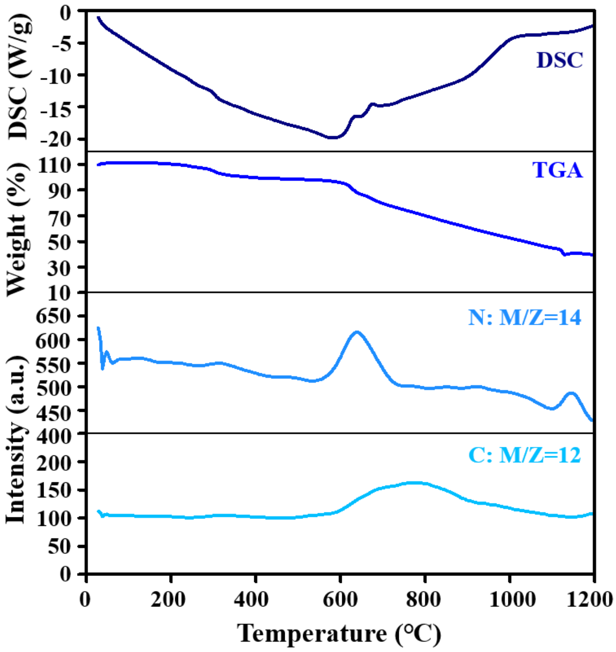

Figure S11. Differential scanning calorimetry, thermal gravimetric analysis of Cu/ZIF, and mass spectrometer analysis of TGA exhaust gas.

An obvious reaction heat flow and significant weight loss were observed at about 590 °C, indicating the transformation from ZIF-8 to N-doped carbon. Moreover, the releasing of reductive elements (N and C) can be observed in the mass spectrometer analysis.

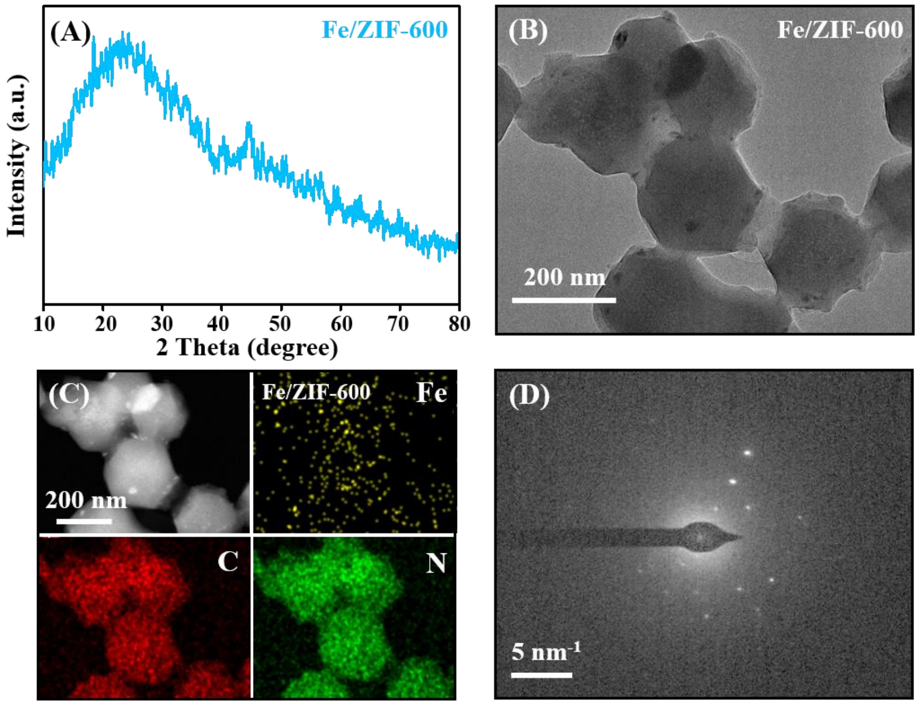

Figure S12. Characterizations of Fe/ZIF-600. (A) XRD pattern. (B, C) TEM image and the corresponding EDS mapping. (D) SAED pattern.

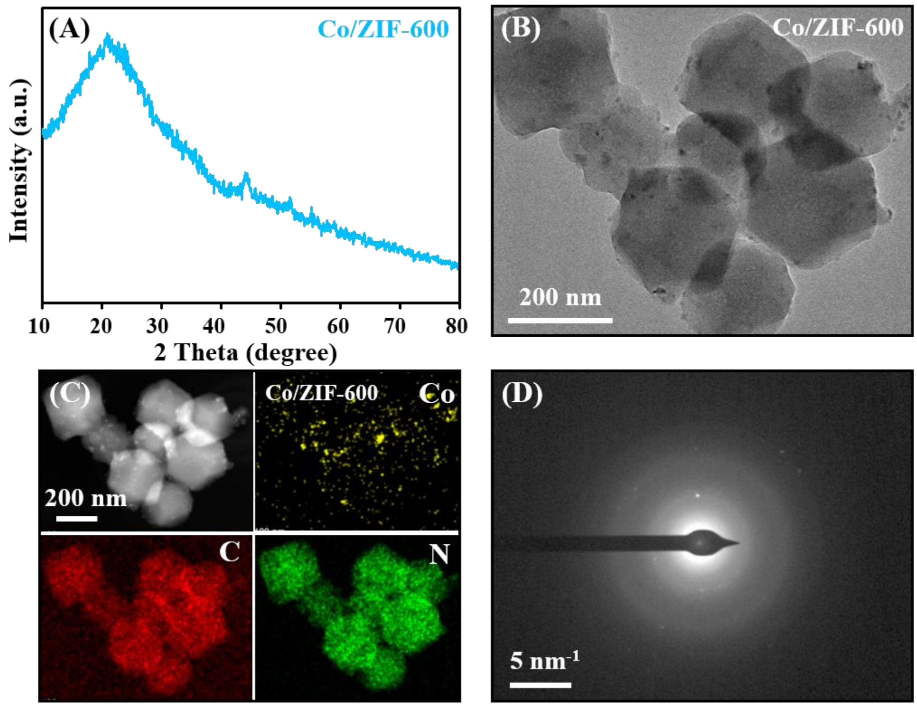

Figure S13. Characterizations of Co/ZIF-600. (A) XRD pattern. (B, C) TEM image and the corresponding EDS mapping. (D) SAED pattern.

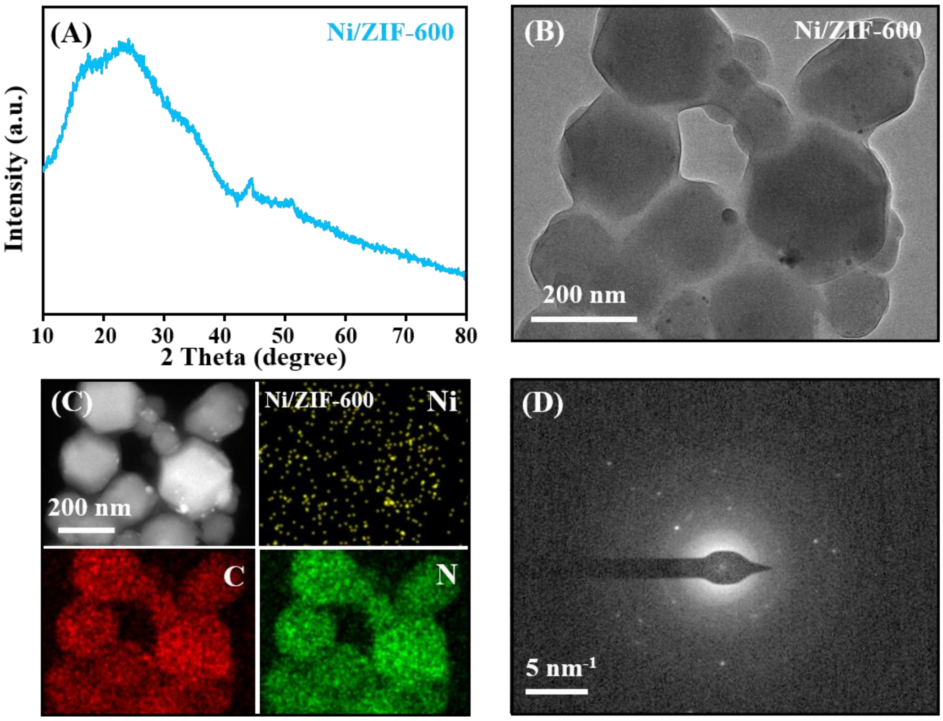

Figure S14. Characterizations of Ni/ZIF-600. (A) XRD pattern. (B, C) TEM image and the corresponding EDS mapping. (D) SAED pattern.

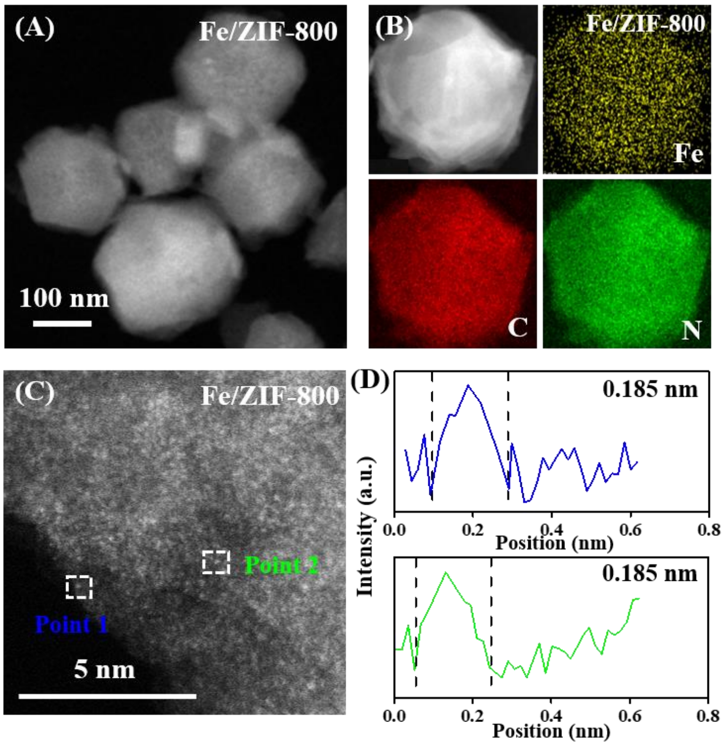

Figure S15. Electron microscopic characterizations of Fe/ZIF-800. (A, B) HAADF-TEM and EDS mapping. (C) HAADF-STEM. (D) The corresponding intensity profiles of selected bright spots.

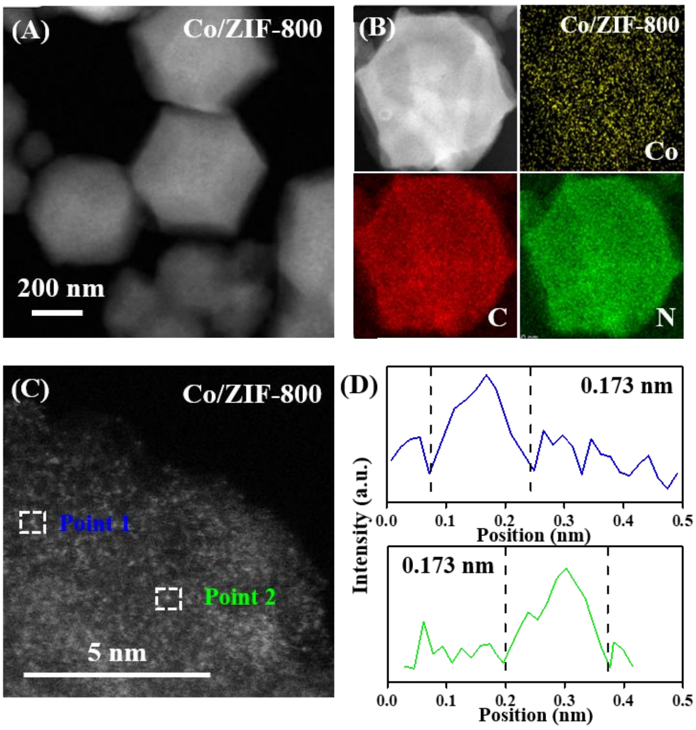

Figure S16. Electron microscopic characterizations of Co/ZIF-800. (A, B) HAADF-TEM and EDS mapping. (C) HAADF-STEM. (D) The corresponding intensity profiles of selected bright spots.

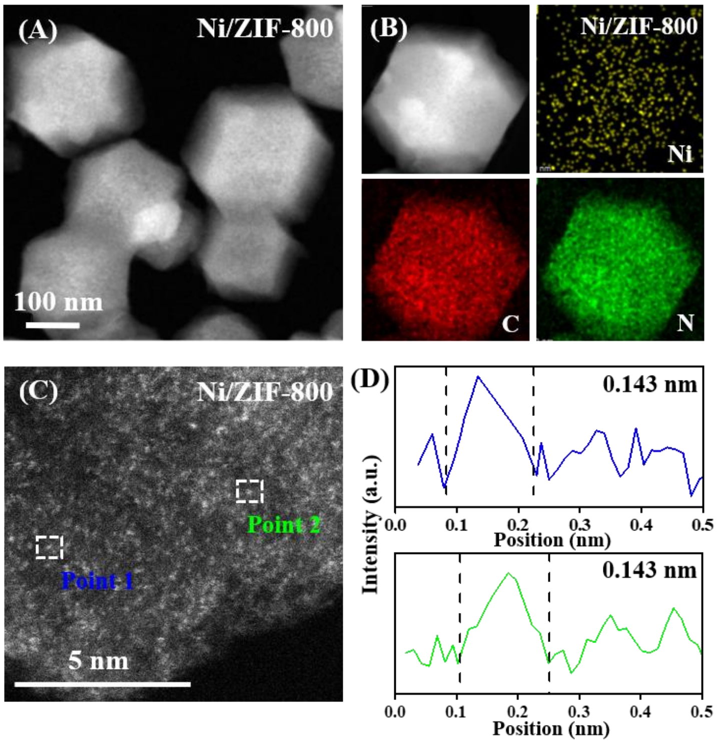

Figure S17. Electron microscopic characterizations of Ni/ZIF-800. (A, B) HAADF-TEM and EDS mapping. (C) HAADF-STEM. (D) The corresponding intensity profiles of selected bright spots.

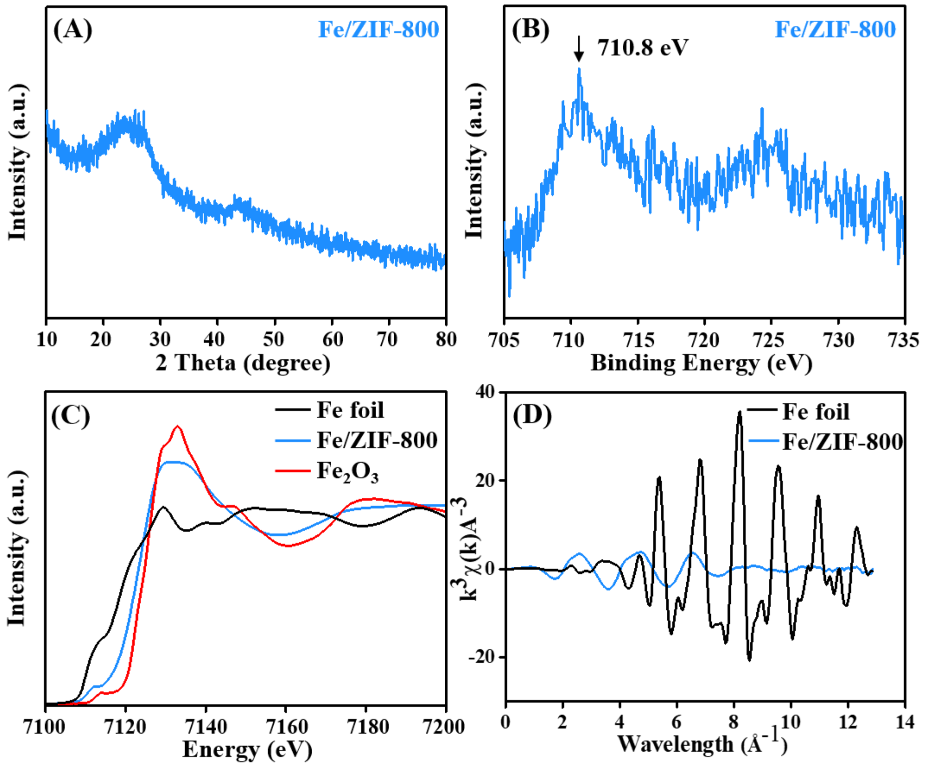

Figure S18. (A) XRD pattern of Fe/ZIF-800. (B) Fe 2p XPS spectrum of Fe/ZIF-800. (C) Fe K edge XANES spectrum of Fe/ZIF-800. (D) Fe K-edge EXAFS (k space plots) for Fe/ZIF-800.

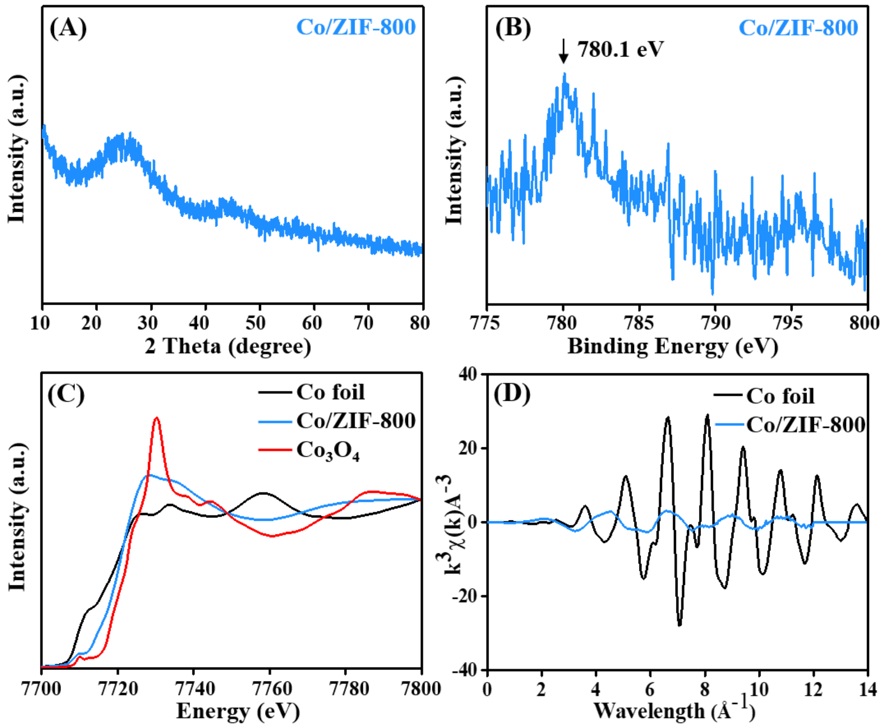

Figure S19. (A) XRD pattern of Co/ZIF-800. (B) Co 2p XPS spectrum of Co/ZIF-800. (C) Co K edge XANES spectrum of Co/ZIF-800. (D) Co K-edge EXAFS (k space plots) for Co/ZIF-800.

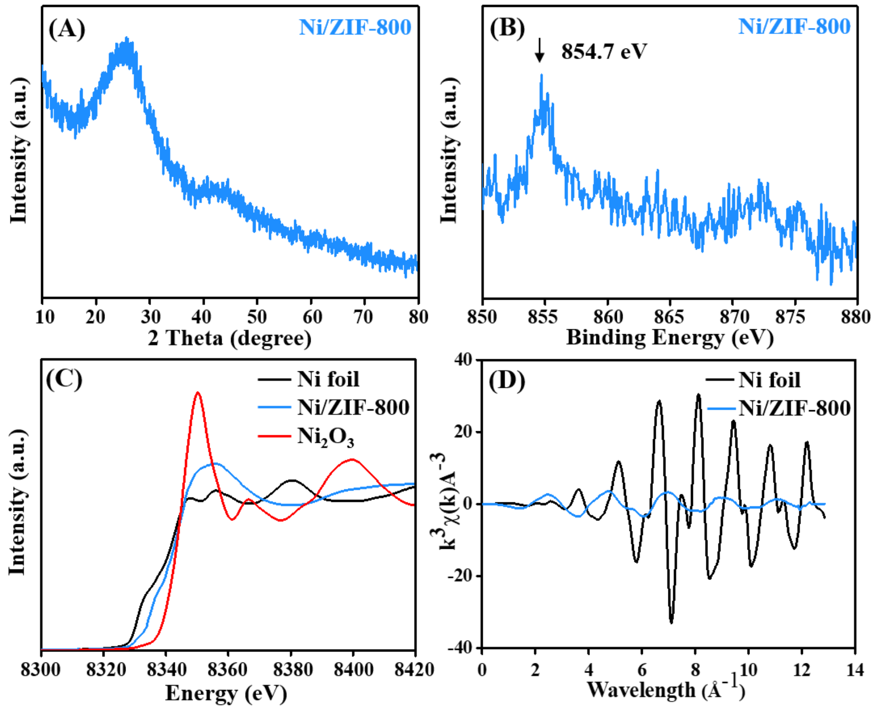

Figure S20. (A) XRD pattern of Ni/ZIF-800. (B) Ni 2p XPS spectrum of Ni/ZIF-800. (C) Ni K edge XANES spectrum of Ni/ZIF-800. (D) Ni K-edge EXAFS (k space plots) for Ni/ZIF-800.

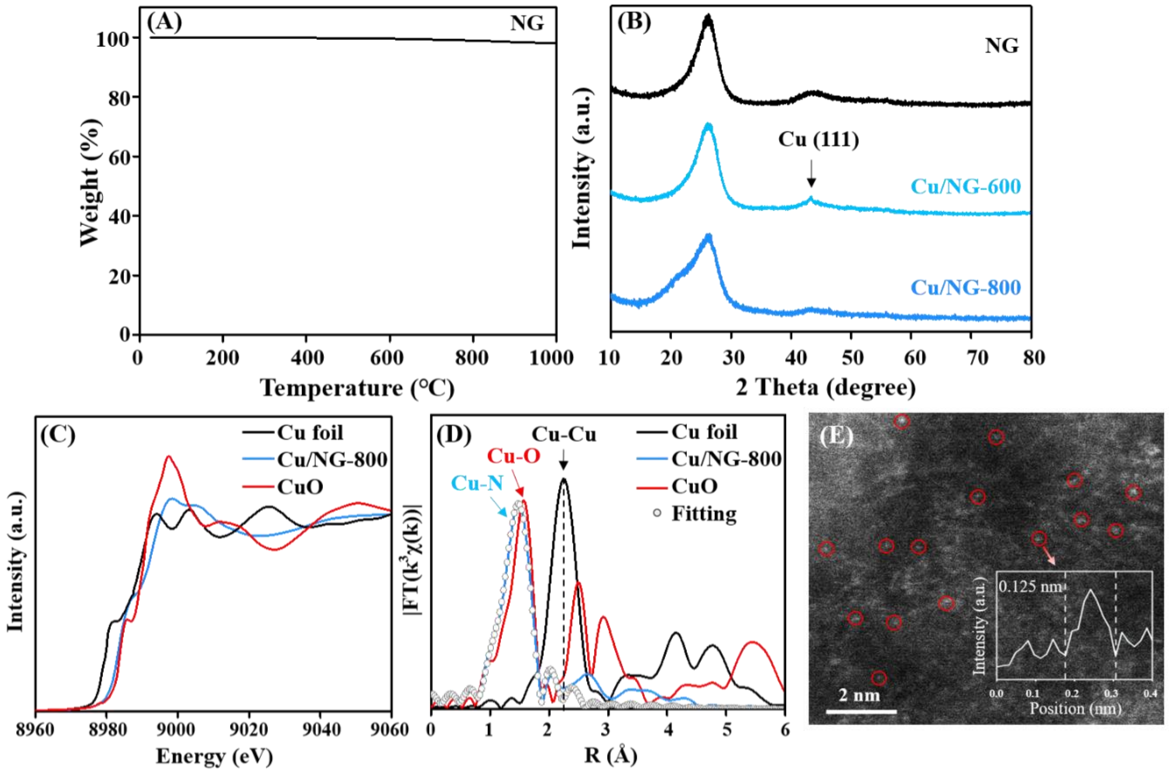

Figure S21. (A) TGA curve of NG. (B) XRD patterns of NG, Cu/NG-600 and Cu/NG-800. (C) Cu K edge XANES spectrum of Cu/NG-800. (D) k^3-weighted χ(k) function of EXAFS spectrum for Cu/NG-800. (E) AC HAADF-STEM image of Cu/NG-800.

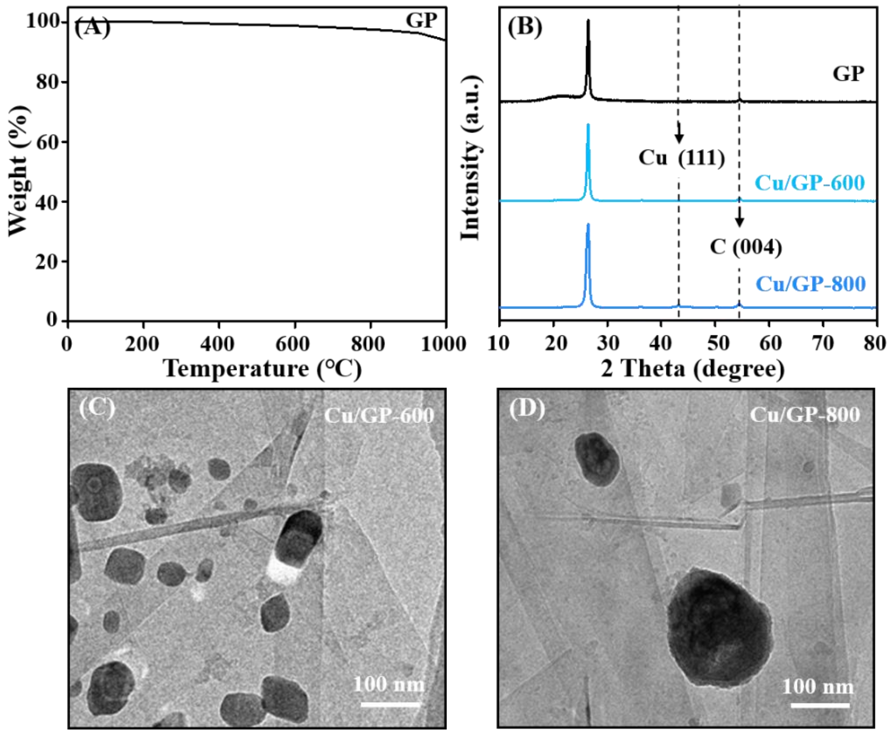

Figure S22. Characterizations of Cu/GP. (A) TGA curve of GP. (B) XRD patterns of GP, Cu/GP-600 and Cu/GP-800. (C, D) TEM images of Cu/GP-600 and Cu/GP-800.

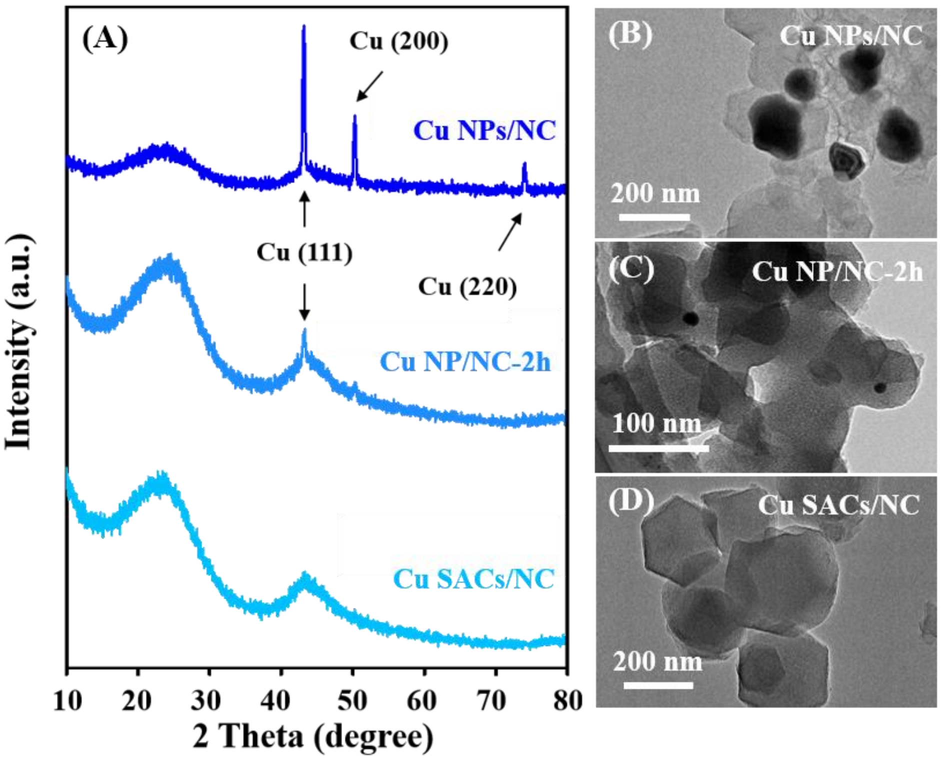

Figure S23. (A) XRD patterns of Cu NPs/NC, Cu NP/NC-2h and Cu SACs/NC. (B-D) TEM images of the Cu NPs/NC, Cu NP/NC-2h and Cu SACs/NC.

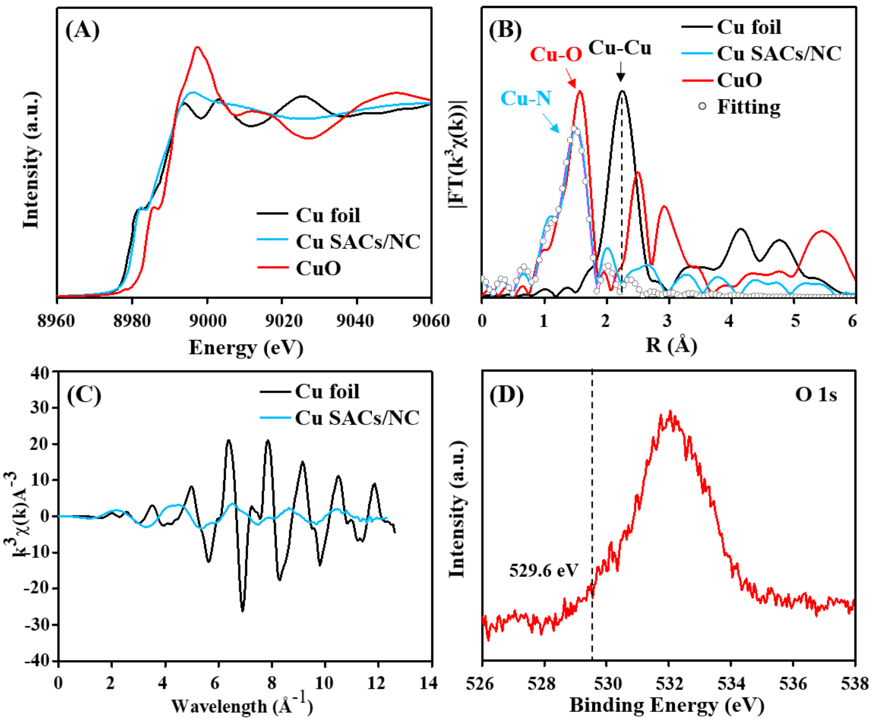

Figure S24. Cu K edge XAFS spectrum of Cu SACs/NC. (A) Cu K edge XANES spectrum. (B) k^3-weighted $\chi(k)$ function of EXAFS spectrum. (C) Cu K-edge EXAFS (k space plots). (D) O 1s XPS spectrum of Cu SACs/NC.

Cu-O bond situated at 529.6 eV[7] was not observed in the O 1s XPS spectrum of Cu SACs/NC, confirming that Cu single atoms are mainly coordinated with nitrogen.

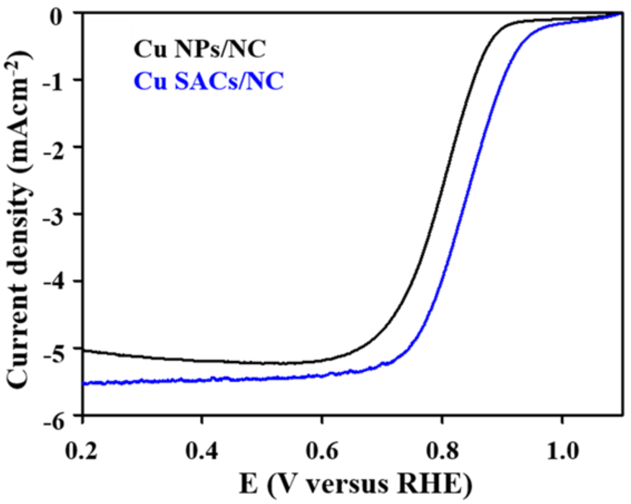

Figure S25. Polarization curves of Cu NPs/NC and Cu SACs/NC in ORR application.

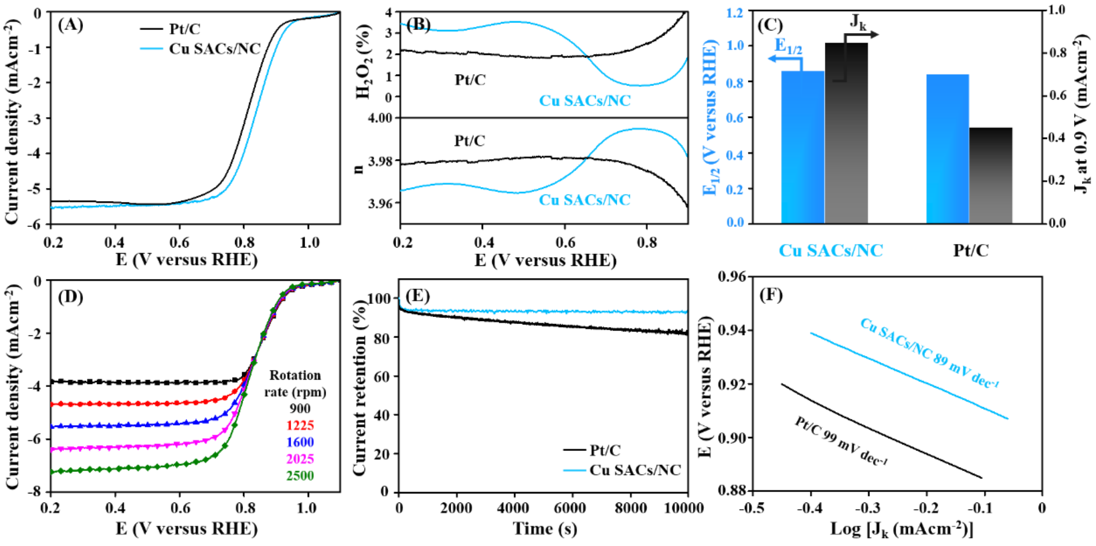

Figure S26. ORR performance of Cu SACs/NC. (A) Polarization curves of Pt/C and Cu SACs/NC. (B) electron transfer number (n, bottom) and H_2O_2 yield (top) versus potential for Cu SACs/NC and Pt/C. (C) Half-wave potentials ($E_{1/2}$) and kinetic currents (Jk at 0.9V) of Cu SACs/NC and Pt/C. (D) RDE polarization curves of Cu SACs/NC at different rotation rates. (E) The i-t chronoamperometric response of Cu SACs/NC and Pt/C. (F) Tafel plots of Cu SACs/NC and Pt/C.

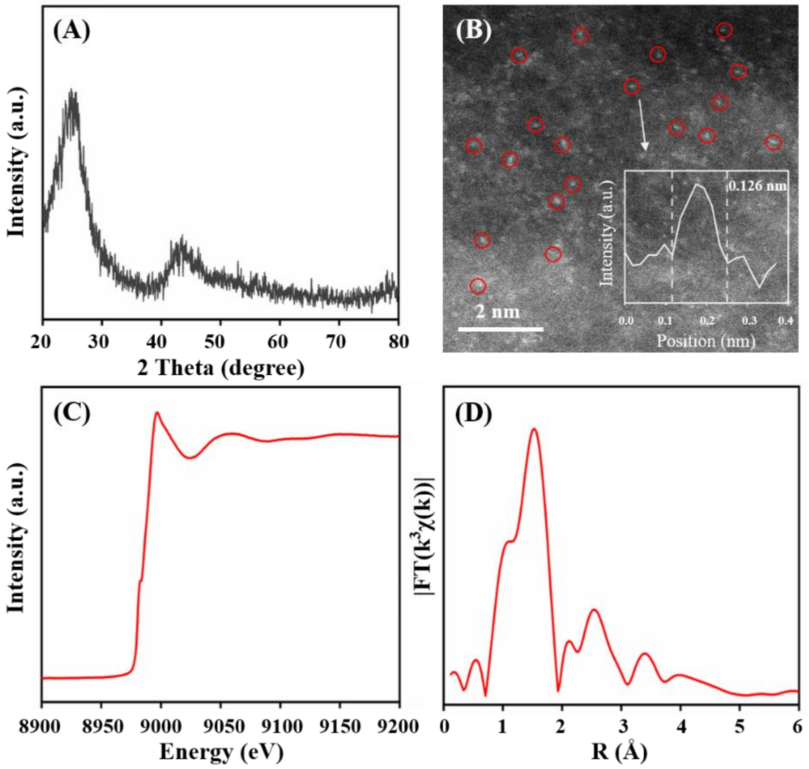

Figure S27. Characterization of the Cu SACs/NC after long-term ORR test. (A) XRD pattern. (B) AC HAADF-STEM image. (C) Cu K edge XANES spectrum. (D) k^3-weighted χ(k) function of EXAFS spectrum.

Table S1. Structural parameters extracted from the Cu K-edge EXAFS fitting.

	Shell	N[a)]	R(Å)[b)]	σ^2 (Å^2·10^{-3})[c)]	ΔE_0 (eV)[d)]	R factor (%)[e)]
Cu/ZIF-800	Cu-N	3.9	1.94	6.5	3.4	0.2
Cu/NG-800	Cu-N	3.8	1.94	7.4	6.5	0.1
Cu SACs/NC	Cu-N	2.7	1.92	7.5	5.1	0.4

a) coordination number; b) bond distance; c) Debye-waller factors; d) the inner potential correction; e) goodness of fit.

$S0^2$ was set as 0.84 for Cu-N, which was obtained from the experimental EXAFS fit of reference CuPc by fixing CN as the known crystallographic value and was fixed to all the samples.

3. References

[1] G. Kresse, J. Furthmüller. *Comput. Mater. Sci.* **1996**, *6*, 15.

[2] G. Kresse, J. Furthmüller. *Phys. Rev. B.* **1996**, *54*, 11169.

[3] J. Perdew, K. Burke, M. Ernzerhof. *Phys. Rev. Lett.* **1996**, *77*, 3865.

[4] G. Kresse, D. Joubert. *Phys. Rev. B.* **1999**, *59*, 1758.

[5] P. Blöchl. *Phys. Rev. B.* **1994**, *50*, 17953.

[6] S. Grimme, J. Antony, S. Ehrlich, H. Krieg. *Chem. Phys.* **2010**, *132*, 154104.

[7] W. Chen, H. Jin, F. He, P. Cui, C. Cao and W. Song, *Nano Res.* **2022**, *15*, 3017.

Author Contributions

Y. Wang and Y. Qu conceived the idea, analyzed the experimental data and co-wrote the manuscript. Z. Han synthesized samples and carried out characterization and electrochemical measurements. R. Li, B. Jia, D. Li and X. Guo helped with the modification of the paper. L. Zheng helped with the XAFS measurement. K. Leng and J. Zheng helped with the characterization and calculation. J. Bai helped perform the analysis with constructive discussions. All authors contributed to the overall scientific interpretation and edited the manuscript.
